# Biosynthesis of depsipeptides with a 3-hydroxybenzoate moiety and selective anticancer activities involves a chorismatase

**DOI:** 10.1074/jbc.RA119.010922

**Published:** 2020-03-12

**Authors:** Yaoyao Shen, Fan Sun, Liu Zhang, Yijia Cheng, Hongrui Zhu, Shu-Ping Wang, Wei-Hua Jiao, Peter F. Leadlay, Yongjun Zhou, Hou-Wen Lin

**Affiliations:** ‡ Research Center for Marine Drugs, State Key Laboratory of Oncogenes and Related Genes, Department of Pharmacy, Ren Ji Hospital, School of Medicine, Shanghai Jiao Tong University, Shanghai 200127, China; § Department of Biochemistry, University of Cambridge, Cambridge CB2 1GA, United Kingdom

**Keywords:** natural product biosynthesis, secondary metabolism, antibiotics, anticancer drug, enzyme catalysis, anticancer drug, chorismatase, non-ribosomal peptide synthetase (NRPS), polyketide synthase (PKS), unantimycins

## Abstract

Neoantimycins are anticancer compounds of 15-membered ring antimycin-type depsipeptides. They are biosynthesized by a hybrid multimodular protein complex of nonribosomal peptide synthetase (NRPS) and polyketide synthase (PKS), typically from the starting precursor 3-formamidosalicylate. Examining fermentation extracts of *Streptomyces conglobatus*, here we discovered four new neoantimycin analogs, unantimycins B–E, in which 3-formamidosalicylates are replaced by an unusual 3-hydroxybenzoate (3-HBA) moiety. Unantimycins B–E exhibited levels of anticancer activities similar to those of the chemotherapeutic drug cisplatin in human lung cancer, colorectal cancer, and melanoma cells. Notably, they mostly displayed no significant toxicity toward noncancerous cells, unlike the serious toxicities generally reported for antimycin-type natural products. Using site-directed mutagenesis and heterologous expression, we found that unantimycin productions are correlated with the activity of a chorismatase homolog, the *nat-hyg5* gene, from a type I PKS gene cluster. Biochemical analysis confirmed that the catalytic activity of Nat-hyg5 generates 3-HBA from chorismate. Finally, we achieved selective production of unantimycins B and C by engineering a chassis host. On the basis of these findings, we propose that unantimycin biosynthesis is directed by the neoantimycin-producing NRPS–PKS complex and initiated with the starter unit of 3-HBA. The elucidation of the biosynthetic unantimycin pathway reported here paves the way to improve the yield of these compounds for evaluation in oncotherapeutic applications.

## Introduction

**Figure 1.**
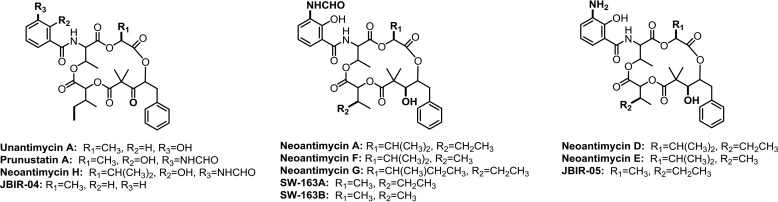
**The structures of neoantimycin derivatives isolated from actinomycete species.**

Antimycin-type depsipeptides are a family of natural products with various important biological activities such as anticancer, antifungal, and anti-inflammatory activities (1). They share a 9-, 12-, 15-, or 18-membered macrolide ring with an amide linkage to a conserved 3-formamidosalicylate (3-FAS)^3^ moiety. In particular, neoantimycins (NATs) feature a 15-membered tetralactone core conjugated with a 3-FAS moiety. The twelve NAT entities previously isolated from actinomycete species differ structurally through varying size of alkyl side chains at the C4 and C9 positions, either hydroxyl or ketone at C1, and the substitution of the conserved 3-FAS with a 3-amidosalicylate, benzoyl, or 3-hydroxybenzoyl (1) (Fig. 1). The majority of NATs show excellent anticancer activities, *e.g.* NAT-A, -F, and -H have IC_50_ values of 0.1–0.8 μm against human ovarian cancer cells (2) and IC_50_ values of 0.2–0.6 μm against human colon cancer cells (3), respectively. In molecular pharmacological studies, the NAT analogs prunustatin A, JBIR-04, and JBIR-05 have been shown to down-regulate GRP-78, a molecular chaperone contributing to chemotherapy resistance (4, 5). Moreover, the 3-FAS moiety in NATs was verified as a potent pharmacophore of inhibitory activity toward oncogenic K-Ras (3).

The biosynthesis of antimycin-type depsipeptides is accomplished by a hybrid complex of NRPS and PKS multimodular proteins, which acts as an assembly line and is typically initiated with the starter unit of 3-FAS (1, 6). The 3-FAS precursor is supplied by a biosynthetic pathway encoded by a gene cassette conservatively associated with the biosynthetic gene clusters (BGCs) of antimycin-type depsipeptides (7, 8). The macrolide skeleton of NATs is generated from a NRPS-PKS multienzyme assembly line by loading the starter unit of 3-FAS and the elongation units of one l-threonine, three α-keto acids, and one malonic acid (2, 9, 10). The offloaded 15-membered macrocyclic rings are converted into the final NATs via C1 hydroxylation conducted by a NAD(P)H-dependent ketoreductase (2).

As the representative moiety in antimycin-type depsipeptides, 3-FAS has been shown to be essential to the anticancer activities of NAT derivatives (3, 11). The 3-FAS group is, however, also known to be mostly responsible for the tight binding of antimycin to the quinone reduction site of the cytochrome *bc*_1_ complex, resulting in inhibition of the mitochondrial respiratory chain (12). Therefore the 3-FAS group could be problematic in development of this family of molecules toward antitumor application.

**Figure 2.**
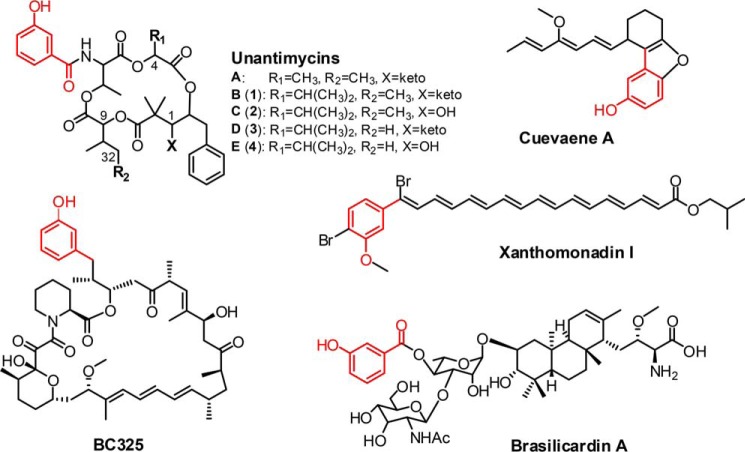
**The structures of the natural products requiring 3-HBA as a building block in their biosynthetic pathways.**

During purification of NATs from the fermentation extract of *Streptomyces conglobatus*, we have discovered four new NAT analogs, unantimycins B–E (**1–4**), which are derivatives of the reported unantimycin A (13) bearing an alternative side chain of 3-hydroxybenzoate (3-HBA) instead of 3-FAS (Fig. 2). Surprisingly, despite replacement of the 3-FAS pharmacophore, **1–4** still exhibited similar levels of anticancer activity compared with cisplatin. Moreover, they mostly presented no obvious toxicities to normal cells. The promising anticancer activities and the obscure biosynthetic origin of unantimycins prompted us to investigate further the biosynthetic pathway. It has been documented that 3-HBA generated by chorismatase from chorismate can be recruited as a starting precursor in the biosynthesis of polyketides BC325, cuevaene, and xanthomonadin (14–17) or as a donor group in the late modification stage of terpenoid brasilicardin biosynthesis (18) (Fig. 2). With the information, we did genomic mining of *S. conglobatus* and discovered a unique candidate FkbO/Hyg5-type chorismatase gene, *nat-hyg5*, located in an unknown type-I PKS BGC. Inactivation of *nat-hyg5* by specific mutation abolished the production of **1**-**4** in the mutant strain, and introduction of the gene into the NAT heterologous expression made **1**-**4** produced as new components in the system. The recombinant protein of Nat-hyg5 was then demonstrated to have the catalytic ability to produce 3-HBA efficiently from chorismate over a broad pH range. These data strongly support a mechanism in which Nat-hyg5 uses chorismate, the end product of shikimate pathway, to produce 3-HBA as an alternative starter unit in NAT biosynthesis to produce **1**-**4**. The discovery of four new NAT analogs with encouraging potential in anticancer activities and elucidation of their biosynthesis will now allow rational bioengineering of the host strain to improve the production of these analogs.

## Results and discussion

### Discovery and structural elucidation of 1–4

Compounds **1**-**4** were originally discovered from *S. conglobatus*, which produces the macrodiolide polyketide conglobatin as the main product (19). To simplify the purification process, **1–4** were isolated from the fermentation extract of RJ2, a mutant strain losing conglobatin production (see “Materials and methods”). To isolate NAT derivatives, a 24-liter fermentation broth was extracted with ethyl acetate, and the extract was purified by consecutive chromatographic fractionation to produce compounds **1** (16.0 mg), **2** (3.6 mg), **3** (3.0 mg), and **4** (0.6 mg) as white amorphous solid. The molecular formulae of **1–4** were established as C_35_H_43_NO_11_, C_35_H_45_NO_11_, C_34_H_41_NO_11_, and C_34_H_43_NO_11_, respectively, based on HR-ESI–MS analysis (Table S2).

According to the 1D and 2D NMR (supporting text SII, Figs. S1, S11–S14, and Tables S4–S7), the structures of compounds **1–4** were elucidated as a 15-membered tetraester ring attached with an acylamino-bounded 3-hydroxybenzoate (3-HBA) moiety and a C2 monosubstituted benzene ring, bearing a C1 ketone for **1** and **3** and a C1 hydroxy for **2** and **4** (Fig. 2). **3** or **4** differ from **1** or **2** at C32 by having a methyl in the former and an ethyl in the latter (Fig. 2). The structures of **1–4** were also confirmed by HR-MS/MS analysis (supporting text SII, Fig. S2, and Table S3). Considering the structural similarities of **1–4** to the documented unantimycin A (13), bearing C4-methyl in contrast to C4-isopropyl of the formers, compounds **1–4** were designated as unantimycin B to E, respectively.

### Anticancer activity evaluation of 1–4

Because the 3-FAS moiety has been proposed to be the pharmacophore of anticancer activities in NATs (3, 11), we wished to know whether compounds **1–4** with the alternative 3-HBA moiety, instead of 3-FAS, would retain anticancer activities. The carcinoma cell lines of human lung (PC9), colon (Sw620), and melanoma (A375) were firstly selected to evaluate the activities of them, using as positive control the chemotherapeutic drug cisplatin. In the bioactivity assay, **1–4** exhibited considerable anticancer activities with IC_50_ values 1.6–8.2 μm, comparable with the IC_50_ of 1.1–6.3 μm detected for cisplatin (Table 1). This is consistent with the activities previously shown for unantimycin A, which structurally differs from **1** at the C4 alkyl group and which has shown moderate cytotoxicity (IC_50_ = ∼10 μm) against human cervix epidermoid carcinoma, promyelocytic leukemia, and sarcoma cell lines (13). Notably, in addition to the exception of **3** showing IC_50_ 8.5 μm to 16HBE cells, **1–4** generally displayed no obvious toxicity (IC_50_ > 31.9 μm) toward the related normal cells, in contrast to the IC_50_ of 2.7–4.6 μm detected for cisplatin (Table 1). Further work will be required to establish convincingly that the apparently selective anticancer activities of **1–4** are endowed by the absence of binding to the quinone reduction site of the cytochrome *bc*1 complex, which makes antimycin A lethal to the mitochondrial respiratory chain (12, 20). Moreover, the broad-spectrum anticancer activities of **1–4** were evaluated by using six other carcinoma cell lines (Table S9). Except for displaying the IC_50_ of 2.3–7.7 μm toward another lung cancer cells A549, these compounds have relatively weak inhibitory effect on other five cell lines such as colorectal cancer cells HT29 (14.9–19.4 μm), ileocecal cancer cells HCT-8 (9.0–17.9 μm), gastric carcinoma cells SGC7901 (6.6–11.9 μm), liver carcinoma cells HepG2 (13.4–18.3 μm), and cervical cancer cells HeLa (15.3–23.6 μm).

**Table 1:** **The cytotoxic activities (IC_50_) of 1–4** The values shown are the means ± S.D. of triplicate determinations.

	PC9*^a^*	16HBE*^b^*	Sw620*^c^*	NCM460*^d^*	A375*^e^*	HaCaT*^f^*
	μ*m*	μ*m*	μ*m*	μ*m*	μ*m*	μ*m*
**1**	2.44 ± 0.19	98.4 ± 1.9	1.62 ± 0.32	65.5 ± 1.5	5.73 ± 0.20	75.8 ± 0.5
**2**	2.80 ± 0.07	31.9 ± 0.8	4.52 ± 0.11	>100	5.05 ± 0.48	>100
**3**	3.50 ± 0.85	8.47 ± 0.12	8.19 ± 0.55	>100	4.84 ± 0.01	>100
**4**	4.47 ± 0.10	76.2 ± 2.8	4.72 ± 0.86	>100	6.78 ± 0.41	>100
Cisplatin	1.07 ± 0.02	3.73 ± 0.06	2.87 ± 0.06	4.59 ± 0.27	6.31 ± 0.40	2.74 ± 0.06

*^a^* Lung cancer cells.

*^b^* Bronchial epithelial cells

*^c^* Colorectal cancer cells.

*^d^* Colonic epithelial cells.

*^e^* Melanoma cells.

*^f^* Epidermal cells immortalized.

### Inactivation of nat-hyg5 gene by site-directed mutation

**Figure 3.**
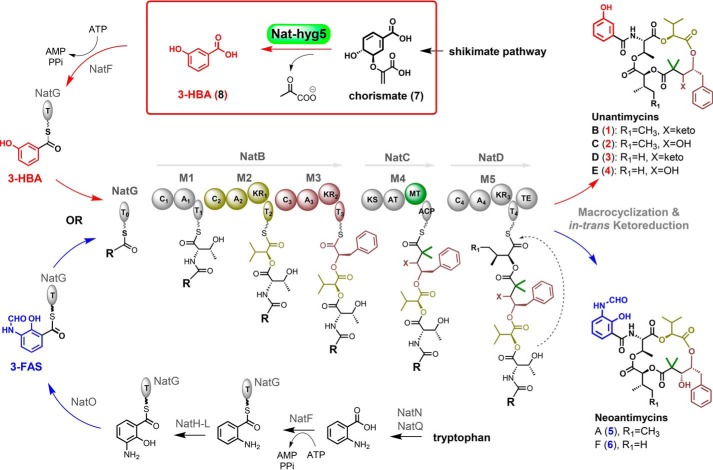
**Proposed biosynthetic pathway of 1**-**4.** The 3-HBA generated by Nat-hyg5 from chorismate, the end product of shikimate pathway, could be accepted as a starter unit to produce **1**-**4** by neoantimycin NRPS-PKS, which originally uses 3-FAS as starting precursor to produce **5** and **6** (2, 9). *A*, adenylation; *T*, thiolation; *C*, condensation; *KR*, ketoreductase; *KS*, ketosynthase; *AT*, acyltransferase; *MT*, methyltransferase; *ACP*, acyl-carrier protein; *TE*, thioesterase. The dimethyl extender unit in M4 was confirmed to be generated by the MT domain acting twice upon a malonate unit loaded by the AT domain (9).

The attachment of 3-HBA moiety in compounds **1**-**4** suggests that the PKS-NRPS assembly line of NAT biosynthesis could accept 3-HBA, instead of 3-FAS, as a starter unit. It is reported that 3-HBA can be generated by CH-Hyg5/CH-II type chorismatase/3-hydroxybenzoate synthase from chorismate, a product of the shikimate pathway (14, 21, 22). However, no homolog of chorismatase was found within or at the boundary of *nat* BGC (Table S8). We therefore conducted genome mining of *S. conglobatus* screening with the sequence of Hyg5 derived from *Streptomyces hygroscopicus*, which has been verified as a CH-Hyg5 type chorismatase producing 3-HBA as the starter unit in the biosynthesis of the rapamycin analog BC325 (14). Interestingly a single candidate gene *nat-hyg5* with a 51% identity (protein sequence) to Hyg5 was discovered, located in an unknown type-I PKS BGC (GenBank^TM^ accession no. MN158725), in which a carboxylic acid ligase domain at the N terminus of a PKS protein (similar to the starting module of rapamycin or FK506 biosynthesis (23)) is likely to load a 3-HBA as starter unit to initiate a polyketide assembly (Fig. S4). We thus postulated that Nat-hyg5 could produce the 3-HBA as an alternative starter unit in NAT biosynthesis to produce **1**-**4** (Fig. 3).

**Figure 4.**
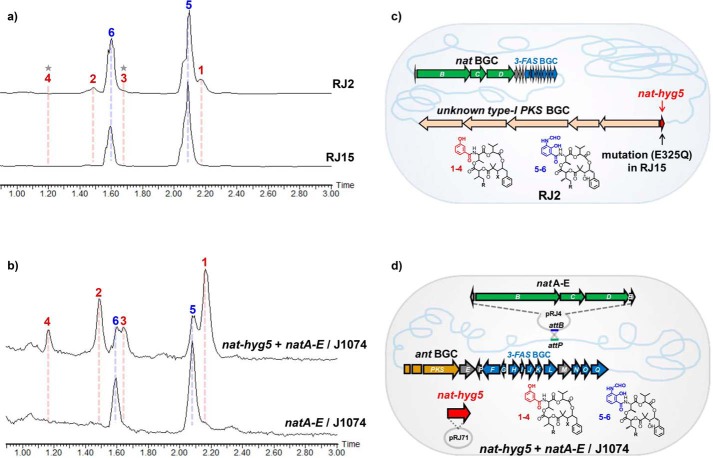
**The production of 1**-**4 is identified to be correlated to the function of *nat-hyg5* gene located in an unknown type-I PKS BGC.** *a*, HPLC-MS analysis of the fermentation extract of RJ2 and the mutant strain RJ15 generated from RJ2 by inactivation of *nat-hyg5* gene (E325Q). *b*, HPLC-MS analysis of the fermentation extract of *S. albus* J1074 containing either pRJ4 (*natA-E*) or pRJ4 and pRJ71 (*natA-E* + *nat-hyg5*). The HPLC-MS data are displayed with the mass extraction of *m*/*z* 664–722. The *stars* labeled in the HPLC-MS of RJ2 represent the elution positions of **3** and **4**, which can be recognized by using specific mass extraction (Fig. S3). *c* and *d*, the graphical schemes of *c* and *d* are corresponding to *a* and *b*, respectively, describing the gene organizations and the expected products of the analyzed strains.

To identify a possible role for the *nat-hyg5* gene in the production of **1**-**4**, we first aimed to inactivate the gene by specific mutation. According to the verified catalysis mechanism for Hyg5, the essential acidic active site residue Glu-334 protonates the methylene group in chorismate to initiate an intramolecular nucleophilic attack and form an arene oxide intermediate, which then undergoes a hydrogen-shift to produce 3-HBA and pyruvate (21). Protein sequence alignment revealed Glu-325 as the residue of Nat-hyg5 corresponding to the active site Glu-334 in Hyg5 (Fig. S6). The active site mutation E325Q was then introduced into the *nat-hyg5* gene in the chromosome of *S. conglobatus* RJ2 through homologous recombination (Fig. 4*c*). As predicted, the resulting mutant RJ15 did not produce **1**-**4** as judged by HPLC-MS analysis (Fig. 4*a*), confirming that *nat-hyg5* is essential for the production of **1**-**4.** This is a rare, but not unprecedented (24, 25), example of cooperation to produce a natural product between a gene cluster and a gene remotely located on the genome, either isolated or within a different BGC.

### Production of 1–4 by co-expression of nat-hyg5 and nat BGC genes in J1074

Having shown that *nat-hyg5* involves in the production of **1**-**4**, we successfully produced these compounds in a heterologous host by co-expression of the *nat-hyg5* gene and the *nat* BGC. We previously established a heterologous system to produce NAT-A (**5**) and -F (**6**) in *Streptomyces albus* J1074 by incorporation of a phiC31 integrative plasmid, pRJ4, containing *natA-E* genes (Fig. 4, *b* and *d*) (2). The metabolite profile of pRJ4/J1074 reveals no production of **1**-**4**, suggesting that no functional 3-hydroxybenzoate synthase homolog exists in J1074. To produce **1**-**4** in the host of pRJ4/J1074, the *nat-hyg5* gene was cloned under the constitutive strong promoter of ermE* (26) using vector ppYJ10 containing the *Streptomyces* replication element derived from pIJ101 (27). The resultant plasmid pRJ71 and the control ppYJ10 were each introduced into pRJ4/J1074 through conjugation. HPLC-MS analysis of the fermentation extract indicated that **1**-**4** were produced as new components after introduction of pRJ71 into pRJ4/J1074, in contrast to the negative control with ppYJ10 introduced instead (Fig. 4*b*). Interestingly, although the production ratio of **1–4** remained same, the yield level of **1–4** was obviously increased relative to that of **5** and **6** in the heterologous expression host by comparing with the parent strain RJ2 (Fig. 4, *a* and *b*), implying that the production of **1**-**4** could be improved in the future by overexpression of *nat-hyg5* for enhancing the supply of 3-HBA precursor. Moreover, the co-appearance of C1 hydroxyl and ketone in **1**-**4** is likely due to the lower activity of the NAD(P)H-dependent ketoreductase NatE toward the substrates of NAT skeleton containing a 3-HBA moiety. This proposal is supported by the *in vitro* assay of NatE with compound **1** as substrate (Fig. S7).

### In vitro characterization of Nat-hyg5

**Figure 5.**
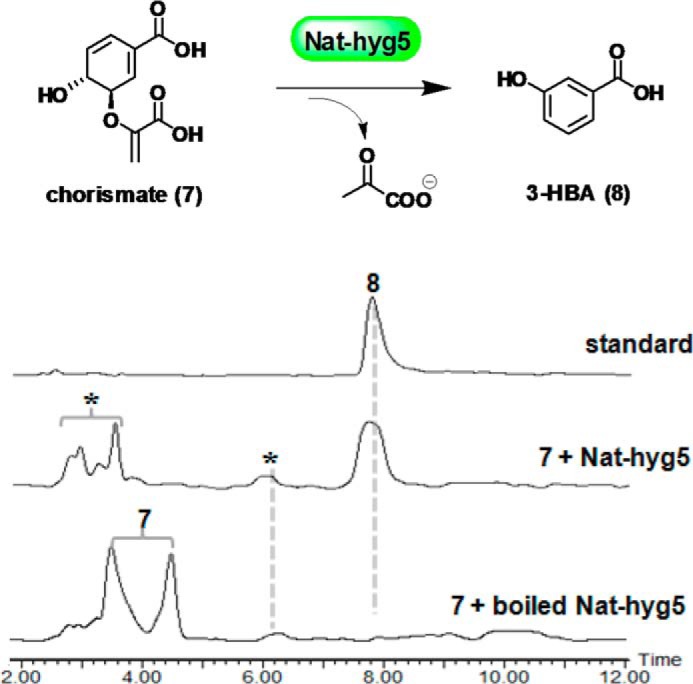
**HPLC-MS analysis of the products generated in Nat-hyg5 enzymatic assays.** The reaction scheme is given at the *top*. HPLC-MS data are displayed with total ion current. *Asterisks* are used to mark the peaks detected in the assay with either intact or boiled Nat-hyg5. The peak at 6.2 min represents 4-HBA. The two peaks labeled ***7*** should represent the isomers of chorismate, which were detected in a reported chorismatase activity assay (14).

To investigate directly the enzymatic activity of *nat-hyg5*, the gene was overexpressed in *Escherichia coli* BL21 (DE3), allowing purification of the encoded Nat-hyg5 protein to near homogeneity. Recombinant Nat-hyg5 with a C-terminal His tag was estimated to have a molecular mass of 37.6 kDa as determined by SDS-PAGE analysis (Fig. S8). Upon incubation of chorismate (4 mm) with Nat-hyg5 (0.5 μm) in Tris-HCl (50 mm, pH 8.0) at 35 °C for 30 min, the expected products 3-HBA and pyruvate were formed as judged by the HPLC-MS analysis, whereas no conversion was observed in the control incubation with boiled Nat-hyg5 (Fig. 5). Chorismate is known to undergo a background spontaneous decomposition to 4-HBA in Tris-HCl buffer at 37 °C (28), and in agreement with this, a trace amount of 4-HBA was detected in the assay with both intact and boiled Nat-hyg5 (Fig. 5). Chorismatases have been classified into four subfamilies: CH-I/CH-FkbO, CH-II/CH-Hyg5, CH-III/CH-XanB2, and CH-IV according to the respective reaction products (21, 22) (Fig. S5). Based on its determined enzymatic activity, Nat-hyg5 should be assigned to the CH-Hyg5/CH-II subfamily (EC 4.1.3.45). Protein sequence alignment confirmed that the characteristic CH-Hyg5/CH-II subfamily residues Gly-164, Gly-231, and Cys-318 are all present in Nat-hyg5 (Fig. S6).

The steady-state kinetic parameters of the Nat-hyg5–catalyzed reaction were determined in a coupled spectrophotometric assay, monitoring the reduction of the pyruvate generated by chorismatase (29). In the assay, Nat-hyg5 was demonstrated to be potently active across a broad pH range, exhibiting *k*_cat_ values of 0.039–0.176 s^−1^ (S.E., 0.001–0.008) over the pH range of 6.5–8.5 (Table 2), in contrast to the two characterized CH-Hyg5/CH-II type chorismatases, Hyg5 (*k*_cat_ 0.22 s^−1^, pH 7.0) and Cuv10 (*k*_cat_ 0.11 s^−1^, pH 6.5) derived respectively from the biosynthetic pathways of the rapamycin analog BC325 and cuevaene A (14, 15). The *K_m_* values of Nat-hyg5 were calculated in the range of 47–65 μm (S.E., 4–8) (Table 2), compared with the 530 μm of Hyg5 and 26 μm of Cuv10 (14, 15). It is worthy of mention that all the kinetic parameters were calculated based on the substrate concentration of 50–300 μm (Fig. S9), and in the lower substrate concentration, no qualified linear value of the enzymatic velocity curve was obtained in this method. The robust activity of Nat-hyg5 suggests a potential application in synthetic biology to biosynthesize the valuable compounds requiring 3-HBA as precursor.

**Table 2:** **The calculated kinetic parameters of Nat-hyg5 in enzymatic assay**

	pH values of assay buffer			
	6.5	7.4	8.0	8.5
*k*_cat_ (×10^−3^ s^−1^)*^a^*	176 ± 8	133 ± 5	84 ± 3	39 ± 1
*K_m_* (μm)*^a^*	47 ± 8	65 ± 8	54 ± 6	50 ± 4
*k*_cat_/*K_m_* (×10^−3^ s^−1^ μm^−1^)*^b^*	3.8	2.1	1.6	0.8

*^a^* The values shown are the means ± S.E. calculated by Prism7 across three replicates.

*^b^* The values shown are the means of three replicates.

### Target accumulation of 1 and 2 in the engineered J1074

To create a clearer background for specific biosynthesis of compounds **1**-**4** in a heterologous host, we set out to generate an engineered J1074 strain solely providing 3-HBA precursor and losing the ability of 3-FAS biosynthesis. To this end, a mutant RJ16 has been generated from J1074 by using a CRISPR-Cas9 system (see “Materials and methods”). In the mutant, the genes *antH–L*, encoding the phenylacetate-CoA oxygenase complex in 3-FAS biosynthesis (7, 30, 31), were replaced by a cassette consisting of *nat-hyg5* gene and two well-characterized strong promoters, A35*p and A26*p (32) (Fig. S10*a*). The two exogenous promoters were designed to motivate, respectively, the transcription of *nat-hyg5* gene and the *antG-antF* operon encoding acyl carrier protein and acyl-CoA ligase required in the loading stage of antimycin-type NRPS-PKS (Fig. 3) (1, 10). Afterward, the plasmid pRJ4 containing *natA-E* was introduced into RJ16 to generate pRJ4/RJ16. According to HPLC-MS analysis, pRJ4/RJ16 loses ability to produce **5** and **6** but solely accumulates **1** and **2** in a lower level compared with the yield in pRJ71 + pRJ4/J1074 (Fig. S10*b*). Moreover, the compounds **3** and **4** produced with trance level in pRJ71 + pRJ4/J1074 (Fig. 4*b*) were not detected in pRJ4/RJ16. We speculate that it might be due to the limit supply of 3-HBA because only one copy of *nat-hyg5* gene is introduced into RJ16. Future production optimization for **1**-**4** could be carried out by either overexpressing *nat-hyg5* in RJ16 or disrupting 3-FAS biosynthesis in RJ2.

## Conclusions

We have discovered four new neoantimycin analogs, unantimycin B-E (**1**-**4**), attached with unusual 3-HBA moiety instead of the conserved 3-FAS in antimycin-type depsipeptides. Compound **1**-**4** showed a similar level of anticancer activities as the chemotherapeutic drug, cisplatin, in bioactivity assays with human lung cancer, colorectal cancer, and melanoma cells. Noticeably, they mostly did not exhibit obvious inhibitory activities against the corresponding normal cells. Because the 3-FAS group in antimycin A has been identified as the functional group correlated with respiratory chain toxicity, we speculate that the substitution of 3-FAS moiety with 3-HBA may contribute to the reduced toxicity of **1**-**4** toward noncancerous cells. This may be a clue for structure–activity optimization of the family of natural products. By using site-directed mutagenesis and heterologous co-expression, the production of **1**-**4** was identified to be correlated to the function of a 3-hydroxybenzoate synthase/chorismatase gene, *nat-hyg5*, associated with an unknown type I PKS BGC. Biochemical characterization and protein sequence analysis confirmed that the Nat-hyg5 is a CH-Hyg5/CH-II type chorismatase showing efficient catalysis in a broad pH range. Evidently the PKS-NRPS assembly line of neoantimycin biosynthesis can accept 3-HBA as an alternative starter unit to produce **1**-**4**, revealing a cross-talk between unrelated natural product biosynthetic pathways. Finally, specific accumulation of **1** and **2** is achieved by creating an engineered chassis host, facilitating further optimization to serve pharmacological study. The work has expanded the neoantimycin family by discovery of four new analogs with promising anticancer properties. The elucidation of the biosynthetic pathway lays a foundation to optimize the compounds yield for oncotherapy application.

## Materials and methods

### Compound purification

The fermentation broth (24 liters) of RJ2, after adjusting the pH 6 with formic acid, was extracted three times with an equal volume of ethyl acetate. The combined organic phase was concentrated under vacuum to afford 32.7 g of syrup extract, which was then dissolved in 400 ml of methanol and degreased three times with 200 ml of hexane. The methanol phase was concentrated to 27.6 g and then subjected to vacuum LC on silica gel (200–300 mesh) and fractionated by using stepwise elution with dichloromethane-methanol (from 50:1 to 0:1, v/v) to afford nine fractions, E1–E9. Guided by HPLC-MS analysis, E1 (10.2 g) and E2 (2.0 g) were combined and separated again through the same vacuum LC column with the elution of petroleum ether-acetone (5:1 to 0:1, v/v) to give ten fractions, E1A–E1J. Then E1D, E1E, and E1F were combined as one fraction (1.5 g) before being separated through an ODS chromatography column (YMC-Pack Pro C18 RS, 20 × 250 mm, 5 μm) by using medium pressure preparative liquid chromatography in the condition of 15 ml/min, 30–100% acetonitrile/H_2_O (0.1% formic acid) in 4 h, and UV210 to obtain nine fractions E1D1–E1D9. Then E1D7 (63 mg) was purified through a semipreparative column (Waters Xbridge C18, 10 × 250 mm, 5 μm) by using the HPLC condition of 80% methanol/H_2_O (0.1% formic acid), 3 ml/min, and UV210 to afford compound **1** (16.0 mg, *t*_R_ 20 min). E1D6 (72 mg) was purified through the same semipreparative column by using the HPLC condition of 62% acetonitrile/H_2_O (0.1% formic acid), 3 ml/min, and UV210 to yield compound **2** (3.6 mg, *t*_R_ 28 min) and **3** (3.0 mg, *t*_R_ 34 min). Finally, E1D5 (4 mg) was purified through an analytical column (Phenomenex C18, 4.6 × 250 mm, 5 μm) by using the HPLC condition of 56% acetonitrile/H_2_O (0.1% formic acid), 0.8 ml/min, and UV210 to produce compound **4** (0.6 mg, *t*_R_ 37 min). Moreover, 0.9 mg more of compound **4** was isolated from another batch of 24 liters of fermentation for bioactivity assay.

### Anticancer and toxicity activities assay

Anticancer activities were evaluated by using human lung cancer cells PC9, human colorectal cancer cells Sw620, and human melanoma cells A375. Toxicity study was conducted by using three types of normal cells, 16HBE (an immortalized human bronchial epithelial cells), NCM460 (a normal human colon mucosal epithelial cell line), and HaCaT (a spontaneously immortalized keratinocyte cell line from adult human skin). All the cell lines were obtained from the Shanghai Institute of Cell Biology, Chinese Academy of Sciences. The cell cultivation media (Gibco) were supplemented with 10% fetal bovine serum, 100 units/ml penicillin, and 100 μg/ml streptomycin. The cells were maintained in a humidified atmosphere with 5% CO_2_ at 37 °C. Half-maximal inhibitory concentration (IC_50_) was determined by a CCK8 assay (Dojindo, Tokyo, Japan). Briefly, the cells were seeded in 96-well plates at a density of 3 × 10^3^ cells/well and incubated for 24 h at 37 °C. Then the cells were treated with various concentrations of compound and incubated for 72 h. Afterward, 10 μl of CCK8 reagent was added to each well before a further incubation for 0.5–4.0 h at 37 °C. Finally, the absorbance of each well in the 96-well plate was measured at 450 nm using microplate reader.

### Deletion of the congE gene

To generate a mutant strain producing compounds **1–4** with clearer background, the *congE* gene putatively essential to conglobatin biosynthesis (19) was selected to be removed in *S. conglobatus* by an in-frame deletion. To construct a gene cassette for homologous recombination, primers CongE-L-S and CongE-L-A and primers CongE-R-S and CongE-R-A (Table S1) were used to amplify a 1736-bp left arm and a 1788-bp right arm, respectively. The two PCR fragments were assembled, by Gibson assembly (33), with the *E. coli–Streptomyces* shuttle plasmid pRJ2 linearized with XbaI and EcoRI (2). The resulting plasmid pRJ3 was used to transform *S. conglobatus* via conjugation from *E. coli* ET12567 with the helper plasmid pUZ8002. The target mutant was screened by using colony PCR with the primers congE-TF and congE-TR (Table S1) from the hygromycin-sensitive colonies prepared after two rounds of propagation on SFM plates without antibiotics present. The PCR product was sequenced to confirm the identity of the target mutant designated as RJ2.

### Site-directed mutation of nat-hyg5

The conserved active site residue E325 in the *nat-hyg5*–encoded enzyme (Fig. S6) was mutated to Gln-325 to inactivate the gene *in situ*. To construct the required gene cassette for homologous recombination, primers Lhyg5-S and Lhyg5-A and primers Rhyg5-S and Rhyg5-A (Table S1) were used to amplify a 1212-bp left arm and a 1472-bp right arm from the genomic DNA of RJ2, respectively. The two PCR fragments were assembled, by Gibson assembly (33), with pRJ2 linearlized with XbaI and EcoRI (2). The resulting plasmid pRJ90 was used to transform RJ2 via conjugation. The target mutant was screened by the same way as above with the primers Hg-S1 and Hg-A1 (Table S1). The target mutant was further confirmed by sequencing of the PCR product and designated as RJ15.

### Co-expression of nat-hyg5 and natA-E in J1074

The *nat-hyg5* gene was amplified from the genomic DNA of RJ2 with the primers Hyg5-S and Hyg5-A (Table S1) and was cloned under the strong constitutive promoter of ermE* in ppYJ10, which contains a *Streptomyces* replication element derived from pIJ101 (27). Through conjugation, the resultant plasmid pRJ71 was used to transform the strain pRJ4/J1074, which produces compounds **5** and **6** with genes *natA-E* cloned in the plasmid pRJ4 (2). The resultant strain pRJ71 + pRJ4/J1074, which is resistant to both thiostrepton and apramycin, was identified from among the exconjugants by colony PCR with the primers C-ppYJ10-S and C-ppYJ10-A (Table S1).

### Engineering of J1074 as a host to specific accumulation of 1 and 2

The *antH-L* genes of antimycin BGC in J1074 were designed to be replaced with a hyg5 cassette (nat-hyg5_A35*p_*ap*(*r*)_A26*p) containing the *nat-hyg5* gene, two strong promoters A35*p and A26*p (32), and an ampicillin-resistant gene (*ap*(*r*)) (see related primers in Table S1). The *nat-hyg5* gene was amplified from pRJ71. The fragment of A35*p_*ap(r*)_A26*p was amplified from ppYJ10 by using three rounds of PCR. The two homologous arms were amplified from the genomic DNA of J1074. By using Gibson assembly, the above four PCR products were assembled into the hyg5 cassette based on a vector fragment, pUC19 fragment1 amplified from ppYJ10, which contains *E. coli* replication. The hyg5 cassette was digested with HindIII and religated to pUC19 fragment2, which is the extension version of pUC19 fragment 1 with the 25/29-bp franking sequences identical to the HindIII/StuI end of pMWcas9 (34). After EcoRI and XbaI digestion, the extended hyg5 cassette was recovered and introduced into the StuI and HindIII sites of the pMWcas9 derivative, which was loaded with a sgRNA sequence targeting the ORF region of *antH* in antimycin BGC. The resultant plasmid pRJ92 was introduced into J1074 by conjugation, and the target mutant was screened by following a reported procedure (34). The resultant mutant RJ16 was further confirmed by sequencing of the PCR products.

### Nat-hyg5 protein expression and purification

For protein expression of Nat-hyg5 with a C-terminal His tag, the gene *nat-hyg5* was amplified from pRJ71 with the primers hyg5-F and hyg5-A (Table S1), and the PCR fragment was introduced into the NdeI and XhoI sites of pET29a by Gibson assembly. The resultant plasmid pRJ89 was used to transform *E. coli* BL21 (DE3) plysS for protein expression. The overnight culture (1 ml) of the target transformant was transferred into 0.5-liter LB medium containing kanamycin (50 μg/ml) (37 °C, 220 rpm). Isopropyl-β-d-thiogalactopyranoside (0.2 mm) was added to the culture once the *A*_600_ reached 0.6–0.8, followed by further cultivation for 15 h at 22 °C. The cells were recovered by centrifugation (11,325 × *g*, 5 min) and resuspended in buffer A (50 mm Tris-HCl, 300 mm NaCl, pH 7.2) for sonication. The cell lysate was centrifuged (34,925 × *g*, 25 min), and the supernatant was passed through a 0.22-μm filter before loading onto a His-Bind affinity column (1-ml bed volume). The target proteins were eluted by stepwise increases of the concentration of imidazole (10 to 500 mm). Concentration and buffer exchanging of the protein was done by using Amicon Ultra-4 concentrators (Millipore, 10-kDa cutoff). Protein-containing fractions of the eluate were analyzed using Bis-Tris gel SDS-PAGE (4–12%). Protein concentrations were measured by using a NanoDrop 1000 spectrophotometer.

### Nat-hyg5 enzyme assays

The enzymatic assay for qualitative analysis was carried out in 100 μl of Tris-HCl buffer (50 mm, pH 8.0) containing 4 mm chorismate (Sigma) and 0.5 μm Nat-hyg5 at 35 °C for 30 min. A negative control consisted of Nat-hyg5 preboiled at 100 °C for 10 min. The reactions were stopped by adding 400 μl of acetonitrile, and the solution was centrifuged at maximum speed for 5 min before taking 20 μl of the supernatant for HPLC-MS analysis.

The kinetic parameters of the Nat-hyg5–catalyzed reaction were determined by lactate dehydrogenase (LDH)–coupled assay (29) in 50 mm Tris-HCl buffer at the pH values of 6.5, 7.4, 8.0, and 8.5 respectively. The assay was carried out in a 96-well plate and monitored at 340 nm by SpectraMax 190 plate reader. First, chorismate with a concentration between 50 and 300 μm was preincubated with 2.5 units/ml LDH (Sigma) and 0.5 mm NADH until the absorption at 340 nm was stable, to ensure that all extraneous pyruvate was converted into lactate by LDH in the consumption of NADH. Subsequently, Nat-hyg5 was added to the system to 0.5 μm concentration to monitor the rate of decline in absorption at 340 nm over 5 min, which reflected the consumption rate of the pyruvate generated by Nat-hyg5 from chorismate. All the reactions were performed in triplicate. Finally, the enzyme kinetic parameters were calculated by fitting the Michaelis–Menten model in the Prism 7 (GraphPad, San Diego, CA). The *k*_cat_ and *K_m_* values are obtained as means ± S.E. calculated by Prism7. The *k*_cat_/*K_m_* values were acquired as the means of three replicates.

### Media, culture conditions, and strains

TSBY medium (3% tryptone soy broth, 0.5% yeast extract, 10.3% sucrose) was used produce mycelium. SFM agar medium (2% soya flour, 2% d-mannitol, 2% agar, 10 mm MgCl_2_) was used for manipulation of conjugation and mutant screening. For NATs production in RJ2, the seed medium (SGCc) consisted of 3% soybean flour, 5% glucose, 0.5% CaCO_3_, 5 mg/liter CoCl_2_·6H_2_O, and 0.2% (v/v) anti-foam, and the production medium (SGC) was the SGCc recipe omitting CoCl_2_·6H_2_O. For the fermentation of J1074-derivative strains, the seed medium was TSBY, and the production medium (SMG) consisted of 3% soya flour, 1% malt extract, 0.3% CaCO_3_, 0.5% glucose, and 0.1% (v/v) antifoam.

Fermentation was carried out by inoculating 150 ml of medium in a 500-ml conical flask fitted with a metal spring, with 10% (v/v) inoculation of 3-day seed culture and then incubating at 30 °C, 220 rpm for 5 days. For small scale fermentation, 50 ml of medium in a 250-ml conical flask was used. *E. coli* strains were grown in Luria–Bertani (LB) broth (1% tryptone, 0.5% yeast extract, 0.5% NaCl) or LB agar (1.5% agar) at 37 °C with the corresponding antibiotics added.

*S. conglobatus* RJ2 was the WT producer of compounds **1–6**. *S. albus* J1074 served as a heterologous host. *E. coli* DH10B was used for plasmid construction. *E. coli* ET12567 containing helper plasmid pUZ8002 was used as a transitional host for introducing target plasmid into *Streptomyces* strains. The *E. coli* BL21 (DE3) plysS strain was used as the host for protein expression.

### DNA manipulation

The oligonucleotides used in this work are summarized in Table S1. Restriction endonucleases and T4 DNA ligase were purchased from New England Biolabs. Gibson assembly solution was the U-Clone master mix kit (Evomic Science, Sunnyvale, CA). Chemicals were purchased from Sigma–Aldrich. Plasmid DNA was isolated from an overnight culture using a plasmid mini kit (Shanghai Generay Biotech). The PCR template of genomic DNA was prepared by using 10% Chelex 100 resin (Bio-Rad) solution. PCR amplifications were carried out using Phusion High-Fidelity PCR Master Mix (New England Biolabs) for cloning, or 2× FastTaq Master Mix (Shanghai Bioroot Biotech) for screening.

### Analytical methods

The HPLC-MS analysis was performed on a Waters HPLC coupled with a Waters Acquity QDa detector. The analytical HPLC was fitted with a Waters Xbridge C18 column (250 mm × 4.6 mm, 5 μm). The solvent system consisted of acetonitrile and H_2_O (0.1% formic acid, v/v). The samples were eluted isocratically at a flow rate 0.8 ml min^−1^ with 75% of acetonitrile over 20 min. The mass spectrometer was run in either positive or negative ionization mode, scanning from *m*/*z* 200 to 1250 with capillary (0.80 kV), cone (15.00 V), and source temperature (120 °C). The high-resolution TOF–electrospray ionization–MS spectra were acquired with a Ultra Performance Liquid Chromatography (UPLC) Waters XeVO G2-XS Q TOF mass spectrometer. The mass spectrometer was run in positive ionization mode, scanning from *m*/*z* 100 to 1200. The MS/MS was carried out with a collision energy ramp of 30–40 eV, a scan time of 0.200 s, and an interscan time of 0.014 s. The UPLC was fitted with a Waters Acquity UPLC BEH C18 column (2.1 mm × 50 mm, 1.7 μm). A solvent system of acetonitrile and H_2_O (0.1% formic acid, v/v) was used for isocratic sample elution with 60% acetonitrile over 5 min at a flow rate of 0.4 ml min^−1^. NMR spectra were recorded on an Agilent DD2 600 MHz NMR spectrometer, using DMSO-*d*_6_ as solvent.

### Data availability statement

Nucleotide and protein sequences of the type-I PKS BGC containing *nat-hyg5* gene have been deposited in the NCBI GenBank^TM^ under accession number MN158725. All other data are contained within the manuscript.

## Author contributions

Y. S., F. S., and S.-P. W. data curation; Y. S. formal analysis; Y. S., F. S., L. Z., Y. C., H. Z., and S.-P. W. investigation; Y. S., H. Z., and S.-P. W. methodology; Y. S. and Y. Z. writing-original draft; F. S., S.-P. W., Y. Z., and H.-W. L. funding acquisition; W.-H. J., P. F. L., and Y. Z. writing-review and editing; P. F. L., Y. Z., and H.-W. L. resources; Y. Z. conceptualization; Y. Z. supervision; Y. Z. project administration.

## Supplementary Material

Supporting Information

## Footnotes

3 The abbreviations used are:

3-FAS: 3-formamidosalicylate
PKS: polyketide synthase
NRPS: nonribosomal peptide synthetase
NAT: neoantimycin
3-HBA: 3-hydroxybenzoate
CH: chorismatase
BGC: biosynthetic gene cluster
HPLC: high-performance liquid chromatography
LDH: lactate dehydrogenase
LB: Luria–Bertani
UPLC: Ultra Performance Liquid Chromatography.
