# Supplementary material for: Biosynthesis of depsipeptides with a 3-hydroxybenzoate moiety and selective anticancer activities involves a chorismatase
Source: J Biol Chem. 2020 Mar 12;295(16):5509–18. doi: 10.1074/jbc.RA119.010922 (PMC7170507; doi:10.1074/jbc.RA119.010922)
Supplement: Supporting Information [file supp_295_16_5509__index.html]

Biosynthesis of depsipeptides with a 3-hydroxybenzoate moiety and selective anticancer activities involves a chorismatase — Anticancer depsipeptide biosynthesis requires a chorismatase — Biosynthesis of depsipeptides with a 3-hydroxybenzoate moiety and selective anticancer activities involves a chorismatase — Anticancer depsipeptide biosynthesis requires a chorismatase — Supporting Information 

# Biosynthesis of depsipeptides with a 3-hydroxybenzoate moiety and selective anticancer activities involves a chorismatase

## Supporting Information

- Supporting Information (to be published online) - Supporting Information, for the main section
