# Supplementary material for: Biosynthesis of depsipeptides with a 3-hydroxybenzoate moiety and selective anticancer activities involves a chorismatase
Source: J Biol Chem. 2020 Mar 12;295(16):5509–18. doi: 10.1074/jbc.RA119.010922 (PMC7170507; doi:10.1074/jbc.RA119.010922)
Supplement: Supporting Information [file supp_RA119.010922_155610_4_supp_492033_q73ybc.docx]

**Supporting Information for**

**Biosynthesis of depsipeptides with a 3-hydroxybenzoate moiety and selective anticancer activities involves a chorismatase**

Yaoyao Shen, Fan Sun, Liu Zhang, Yijia Cheng, Hongrui Zhu, Shu-Ping Wang, Wei-Hua Jiao, Peter F. Leadlay, Yongjun Zhou*, Hou-Wen Lin*

* To whom correspondence should be addressed.

E-mail: Dr. Y. Zhou: [zhouyongjun66@163.com](mailto:zhouyongjun66@163.com); Dr. H. W. Lin: [franklin67@126.com](mailto:franklin67@126.com)

**List of the Material Included:**

**SI. Tables S1-S9**

**Table S1**. Oligonucleotides used in this study………………………………………...2

**Table S2**. HR-MS data of compounds **1**-**4**…………………………………………….4

**Table S3**. HR-MS/MS data of compounds **1**-**4**………………………………………..5

**Table S4**. ^1^H (600 MHz) and ^13^C (151 MHz) NMR data for **1** in DMSO-*d*_6_…………6

**Table S5**. ^1^H (600 MHz) and ^13^C (151 MHz) NMR data for **2** in DMSO-*d*_6_…………7

**Table S6**. ^1^H (600 MHz) and ^13^C (151 MHz) NMR data for **3** in DMSO-*d*_6_…………8

**Table S7**. ^1^H (600 MHz) and ^13^C (151 MHz) NMR data for **4** in DMSO-*d*_6_…………9

**Table S8**. The deduced orfs of neoantimycin biosynthetic gene cluster……………..10

**Table S9**. The cytotoxic activities of **1-4** against six other human cancer cell lines…….....11

**SII. Structure Elucidation of Compounds 1-4**……………………………...……..12

**SIII. Figs. S1-S14**

**Fig. S1.** Diagnostic 2D NMR (DMSO-*d_6_*) correlations for compounds **1**-**4**…………13

**Fig. S2**. Identification of compounds **1**-**4** by HR-MS/MS analysis……………….…13

**Fig. S3**. Confirmation of the production of **1**-**4** in the fermentation extract of RJ2…15

**Fig. S4**. Annotation of the *nat-hyg5* gene associated type-I PKS gene cluster………16

**Fig. S5**. The reaction products of the four chorismatase subfamilies…………..……16

**Fig. S6**. Protein sequence alignment of the Nat-hyg5 homologues……………….…17

**Fig. S7**. The partial activity of NatE with compound **1** as substrate…………………18

**Fig. S8**. SDS-PAGE gel of Nat-hyg5 protein……………………………………...…19

**Fig. S9.** The Michaelis-Menten plots of Nat-hyg5-catalyzed reactions…………...…19

**Fig. S10.** Target accumulation of compounds **1** and **2** in RJ16…………………....…20

**Fig. S11**. NMR spectra of compound **1**…………………………………………....…21

**Fig. S12**. NMR spectra of compound **2**…………………………………………...…25

**Fig. S13.** NMR spectra of compound **3**…………………………………………...…29

**Fig. S14.** NMR spectra of compound **4**…………………………………………...…33

**SI. Tables S1-S9**

**Table S1**. Oligonucleotides used in this study

| **Primer** | | **Sequence** **(5’ -> 3’)** | | **Description** |
| --- | --- | --- | --- | --- |
| **Generate a homologous recombination cassette in pRJ90 for site-mutation of *nat-hyg5*** | | | | |
| Lhyg5-S | | GATCCCCGGGGACCTGCAGGTCGACTTACTCCTCATGCAATACAGACCCGTTGG (the underline: 26 bp overlapping with the XbaI side of pRJ2) | | 1212 bp left arm |
| Lhyg5-A | | TGATGCCCTCGATCTGCACGAGCAGGTCGGAGCGGCAGATGTC | |  |
| Rhyg5-S | | CTGCTCGTGCAGATCGAGGGCATCATCGCCTGACAGAG | | 1472 bp right arm |
| Rhyg5-A | | ATGACATTAACCTATAAAAATAGGCGTATCACGAGGCCCTTTCGTCTTC (the underline: 51 bp overlapping with the EcoR I side of pRJ2) | |  |
| **Identify the target point-mutation in *nat-hyg5*** | | | | |
| Hg-S1 | | AGGCATTCGAGCAGTACAC | | 1229 bp product |
| Hg-A1 | | TGCTCTCACCCTCCATGC | |  |
| **Cloning of *nat-hyg5* gene based on ppYJ10 to generate pRJ71** | | | | |
| Hyg5-S | | TCGTGCCGGTTGGTAGGATCCACATATGCTGCGATGCGATTACATCGTCC  (the underline: 25 bp overlapping with NdeI side of ppYJ10) | | 1046 bp product |
| Hyg5-A | | TTTTCAGTGGTGGTGGTGGTGGTGCTCAGGCGATGATGCCCTCGATCT  (the underline: 25 bp overlapping with XhoI side of ppYJ10) | |  |
| **Identify the presence of pRJ71 or ppYJ10 in J1074** | | | | |
| C-pY10-S | | TTCCATCCCCATGATGAGCCAGAC | | 1439 bp product |
| C-ppYJ10-A | | TTCATTTACTAACGTCTGGAAAGACGAC | |  |
| **Protein expression of Nat-hyg5 with a C-terminal 6×His tag** | | | | |
| hyg5-F | | TGTTTAACTTTAAGAAGGAGATATACAATGCTGCGATGCGATTACATCGTCC (the underline: 27 bp overlapping with NdeI side of pET29a) | | 1044 bp product |
| hyg5-A | | ATCTCAGTGGTGGTGGTGGTGGTGCGCGATGATGCCCTCGATCTC  (the underline: 25 bp overlapping with XhoI side of pET29a) | |  |
| **Construction of *nat-hyg5*_*A35**p_ap (r)_*A26**p cassette in a sub-clone:**  The four fragments were assembled with the pUC19-fragment-1, and then re-located into pUC19-fragment-2 after HindIII digestion. | | | | |
| **fragment-1:** left arm of homologous recombination for *antH-L* knock-out | | | | |
| Ant-L-S | AAGCTTAGGTGTAGGAGAGGGAGACGTCGCGTTC | | | Template: J1074 |
| Ant-L-A | TGATTCCACCCCTTCCCACGAGGAGAGTC | | |  |
| **fragment-2:** right arm of homologous recombination for *antH-L* knock-out | | | | |
| Ant-R-S | ACGGACCTTTGAGGAGGGCTCAATGAGCACCATCAGGGAGTTGCTG | | | Template: J1074, |
| Ant-R-A | ACTTGGTCTGACAGAAGCTTCGATCTCCGAGGGGTAGACGTTCTGC  (the underline: 22 bp and 45 bp overlapping with the neighboring fragments) | | |  |
| **fragment-3:** *nat-hyg5* gene | | | | |
| Anth5-S | ACTCTCCTCGTGGGAAGGGGTGGAATCAGGCGATGATGCCCTCGATCTC | | | Template: pRJ71 |
| Anth5-A | GACAACCTACGAACGAGGAGGCCCCATATGCTGCGATGCGATTACATCGTCC  (the underline: 28 bp and 27 bp overlapping with the fragment-1 and -4) | | |  |
| **fragment-4:** *A35**p-ap (r)-*A26**p cassette generated through three rounds of PCR amplification | | | | |
| Blap-S1 | TGTAGCGTGTATTTCGGGGATGGGGCAAGGGCGGACGCTGATACGCCTATTTTTATAGGTTAATG | | Template: ppYJ10  First round of PCR | |
| Blap-A1 | CTAATCGTGAACCTTCTCGAGCGTCAAGTCATCAGGGTTACCAATGCTTAATCAGTGAGGCAC | |  |  |
| Blap-F2 | TTCGTAGGTTGTCGACACTGTAGCGTGTATTTC | | Template: PCR product from first round | |
| Blap-R2 | TCCGTAACCGGCATGCTAATCGTGAACCTTCTC | |  |  |
| Blap-F3 | AGCATATGGGGCCTCCTCGTTCGTAGGTTGTCGACAC | | Template: PCR product from second round | |
| Blap-R3 | CATTGAGCCCTCCTCAAAGGTCCGTAACCGGCATGCT | |  |  |
| **pUC19-fragment-1** | | | | |
| Ecoli-S | CAGAACGTCTACCCCTCGGAGATCG**AAGCTT**CTGTCAGACCAAGTTTACTCATATATAC (the underline: 26 bp overlapping with fragment-1) | | Template: ppYJ10 | |
| Ecoli-A | ACGCGACGTCTCCCTCTCCTACACCT**AAGCTT**CAGCTGCATTAATGAATCGGC (the underline: 27 bp overlapping with fragment-2) | |  |  |
| **pUC19-fragment-2** | | | | |
| Ecoli-S1 | ATATA**AAGCTT**CCTAAAAAATAGCTTCACGCTGCCGCAA**GAATTC**TTCTGTCAGACCAAGTTTACTC (**Boldface**: HindIII, EcoRI; the underline: 29 bp and 25 bp overlapping with the franking sides of pMWcas9 (StuI, HindIII)) | | Template: ppYJ10 | |
| Ecoli-A1 | AATAT**AAGCTT**TGCATGCCTGCAGGTCGACGGATC**TCTAGA**TCAGCTGCATTAATGAATCG (**Boldface**: HindIII, XbaI) | |  |  |
| **Cloning of 20 bp single guide RNAs (sgRNA) in pMWcase9 (EcoRI+XbaI)** | | | | |
| Antsg-S1 | AATTCGAGCGCGGGCACTACGACTTGTTTTAGAG | | The overhang sequences of annealed oligos are complementary to the ends of pMWcas9 (EcoRI, XbaI) | |
| Antsg-A1 | CTAGCTCTAAAACAAGTCGTAGTGCCCGCGCTCG  (underline: 20 bp sgRNA sequence) | |  |  |
| **Identification of *natH-L* knock-out and the introduction of *nat-hyg5*_*A35**p_ap (r)_*A26**p cassette in J1074** | | | | |
| C-ant-S | TCAGGCTCAGGACACGCTTCT | | 2014 bp product.  Check the recombination location of left arm | |
| C-ant-A2 | ACAGCATCGACAAGCAGTGCCA | |  |  |
| Lan5-S | TCCAGGGACCGCTGGACGATG | | 1379 bp product.  Check the presence of *nat-hyg5* | |
| Lan5-A | ACTTCGTCCTGGGGGAGGTCAC | |  |  |
| C-Rant-S | AGATCGCTGAGATAGGTGCCTCAC | | 1991 bp product.  Check the recombination location of right arm | |
| C-Rant-A | TCATCCACGACAACCAGCACC | |  |  |
| **In-frame deletion of *congE* gene in conglobatin gene cluster (GenBank: LN849060.1)** | | | | |
| CongE-L-S | ATCCCCGGGGACCTGCAGGTCGACtctagaGTGCTGCTGGGCGTCATGAAGTC (the underline: 25 bp overlapping with the XbaI side of pRJ2) | | 1736 bp left arm | |
| CongE-L-A | CTCTCGTTCTTTAACATATCTGGCACACGCGCATG | |  |  |
| CongE-R-S | TGCCAGATATGTTAAAGAACGAGAGGCGCAGCAAC | | 1788 bp right arm | |
| CongE-R-A | TATCACGAGGCCCTTTCGTCTTCAAGAATTCCCATCGCGTCGGGACCACCA (the underline: 26 bp overlapping with the EcoRI side of pRJ2) | |  |  |
| **Identify the target deletion of *congE*** | | | | |
| congE-TF | ACCTCGCTGCTGCTGATGCGGCTG | | 1711 bp for wild type  604 bp for target mutant | |
| congE-TR | TGCCGGTGCTGTGGGTCGAGGATC | |  |  |

**Table S2.** HR-MS data of compounds **1**-**4**. (related to Fig. S2)

| **compounds** | **formula** | **calc. *m/z*** | **det. *m/z*** | **error (ppm)** |
| --- | --- | --- | --- | --- |
| **1** | H^+^C_35_H_43_NO_11_ | 654.2909 | 654.2908 | -0.2 |
| **2** | H^+^ C_35_H_45_NO_11_ | 656.3065 | 656.3065 | 0.0 |
| **3** | H^+^C_34_H_41_NO_11_ | 640.2752 | 640.2755 | 0.5 |
| **4** | H^+^C_34_H_43_NO_11_ | 642.2909 | 642.2906 | 0.3 |

**Table S3**. HR-MS/MS data of compounds **1**-**4**. (related to Fig. S2)

| **fragments** | **formula** | **calc. *m/z*** | **related**  **compounds** | **det. *m/z*** | **error (ppm)** |
| --- | --- | --- | --- | --- | --- |
| **F1** | C_10_H_10_NO_2_^+^ | 176.0706 | **1** | 176.0707 | 0.6 |
|  |  |  | **2** | 176.0709 | 1.7 |
|  |  |  | **3** | 176.0710 | 2.3 |
|  |  |  | **4** | 176.0703 | -1.7 |
| **F2** | C_11_H_10_NO_3_^+^ | 204.0655 | **1** | 204.0658 | 1.5 |
|  |  |  | **2** | 204.0660 | 2.5 |
|  |  |  | **3** | 204.0659 | 2.0 |
|  |  |  | **4** | 204.0654 | -0.5 |
| **F3** | C_11_H_12_NO_4_^+^ | 222.0761 | **1** | 222.0763 | 0.9 |
|  |  |  | **2** | 222.0765 | 1.8 |
|  |  |  | **3** | 222.0764 | 1.4 |
|  |  |  | **4** | 222.0757 | -1.8 |
| **F4** | C_16_H_18_NO_5_^+^ | 304.1179 | **1** | 304.1181 | 0.7 |
|  |  |  | **2** | 304.1185 | 2.0 |
|  |  |  | **3** | 304.1183 | 1.3 |
|  |  |  | **4** | 304.1181 | 0.7 |
| **F5** | C_16_H_20_NO_6_^+^ | 322.1285 | **1** | 322.1287 | 0.6 |
|  |  |  | **2** | 322.1291 | 1.9 |
|  |  |  | **3** | 322.1289 | 1.2 |
|  |  |  | **4** | 322.1286 | 0.3 |
| **F6** | C_25_H_30_NO_8_^+^ | 472.1966 | **2** | 472.1972 | 1.3 |
|  |  |  | **4** | 472.1958 | -1.7 |
| **F7** | C_29_H_34_NO_8_^+^ | 524.2279 | **2** | 524.2289 | 1.9 |
|  |  |  | **4** | 524.2278 | -0.2 |
| **F8** | C_29_H_36_NO_9_^+^ | 542.2385 | **2** | 542.2391 | 1.1 |
|  |  |  | **4** | 542.2382 | -0.6 |

| **Table S4**. ^1^H (600 MHz) and ^13^C (151 MHz) NMR data for **1** in DMSO-*d*_6_. | | | | | | |
| --- | --- | --- | --- | --- | --- | --- |
| **No.** | ***δ*_C_** | ***δ*_H_ , mult. (*J* in Hz)** | | **HMBC** | **COSY** | **ROESY** |
| 1 | 202.5 |  | | | | |
| 2 | 76.5 | 5.39, dd (10.1, 2.7) | 1, 3, 12, 13 | | 12a, b | 14/18, 35 |
| 3 | 168.1 |  |  | |  |  |
| 4 | 75.5 | 5.02, d (3.0) | 3, 5, 19, 20, 21 | | 19 |  |
| 5 | 168.5 |  |  | |  |  |
| 6 | 55.4 | 5.15, dd (9.2, 3.0) | 5 | | 6-NH, 7 | 29 |
| 7 | 70.3 | 5.66, qd (6.4, 2.9) | 8, 29 | | 6, 29 |  |
| 8 | 167.8 |  |  | |  |  |
| 9 | 75.6 | 5.21, d (8.2) | 8, 10, 30, 31, 32 | | 30 |  |
| 10 | 170.8 |  |  | |  |  |
| 11 | 55.0 |  |  | |  |  |
| 12 | 36.6 | 3.16, dd (14.8, 2.7)  2.91, dd (14.7, 10.1) | 1, 13, 14/18  1, 2, 13, 14/18 | | 2, 12b  2, 12a | 14/18, 35  14/18 |
| 13 | 136.2 |  |  | |  |  |
| 14/18 | 129.2 | 7.27, m | 12, 16 | | 15/17 | 2, 35 |
| 15/17 | 128.6 | 7.33, m | 13, 16 | | 14/18, 16 |  |
| 16 | 126.9 | 7.25, m | 14/18 | | 15/17 |  |
| 19 | 29.8 | 2.24, m | 3, 20, 21 | | 4, 20, 21 |  |
| 20 | 16.1 | 0.78, d (6.8) | 4, 19, 21 | | 19 |  |
| 21 | 18.3 | 0.90, d (6.9) | 4, 19, 20 | | 19 |  |
| 22 | 167.9 |  |  | |  |  |
| 23 | 135.2 |  |  | |  |  |
| 24 | 114.8 | 7.27, m | 22, 26, 28 | |  | 6-NH, 7 |
| 25 | 157.2 |  |  | |  |  |
| 26 | 118.5 | 6.94, ddd (8.0, 2.5, 1.0) | 24, 25,28 | | 27 |  |
| 27 | 129.2 | 7.27, m | 23, 25 | | 26, 28 |  |
| 28 | 118.5 | 7.33, m | 22, 24, 26 | | 27 |  |
| 29 | 16.2 | 1.21, d (6.5) | 6, 7 | | 7 | 6, 6-NH |
| 30 | 36.7 | 1.81, qd (7.7, 3.8) | 9, | | 9, 31, 32 |  |
| 31 | 13.8 | 0.87, m | 9, 30, 32 | | 30 | 29 |
| 32 | 24.1 | 1.45, ddd (13.8, 7.4, 4.0)  1.07, m | 9, 30, 31 ,33  9, 30, 31, 33 | | 30, 32b, 33  30, 32a |  |
| 33 | 10.6 | 0.87, m | 9, 30, 32 | | 32a | 9 |
| 34 | 20.7 | 1.32, s | 1, 10, 11, 35 | |  | 35 |
| 35 | 21.3 | 1.41, s | 1, 10, 11, 34 | |  | 2, 12a, 14/18 |
| 6-NH |  | 8.71, d (9.2) | 6, 7, 22 | | 6 | 7, 28, 29 |
| 25-OH |  | 9.69, s | 24, 25, 26 | |  |  |

| **Table S5**. ^1^H (600 MHz) and ^13^C (151 MHz) NMR data for **2** in DMSO-*d*_6_. | | | | | |
| --- | --- | --- | --- | --- | --- |
| **No.** | ***δ*_C_** | ***δ*_H_ , mult. (*J* in Hz)** | **HMBC** | **COSY** | **NOESY** |
| 1 | 78.2 | 3.28, d (10.5) | 10, 11, 12, 35 | 1-OH | 2, 12a, 35 |
| 2 | 72.0 | 5.37, dd (10.5, 4.3) | 3, 12 | 12a | 1, 34 |
| 3 | 168.3 |  |  |  |  |
| 4 | 75.7 | 5.27, d (3.4) | 5, 19, 20, 21 | 19 |  |
| 5 | 168.4 |  |  |  |  |
| 6 | 55.7 | 5.04, dd (9.1, 3.4) | 5, 7, 22 | 6-NH, 7 | 29 |
| 7 | 71.2 | 5.49, qd (6.4, 3.4) | 5, 8 | 6, 29 |  |
| 8 | 168.1 |  |  |  |  |
| 9 | 74.6 | 4.56, d (8.6) | 8, 10, 30, 31, 32 | 30 |  |
| 10 | 175.5 |  |  |  |  |
| 11 | 45.5 |  |  |  |  |
| 12 | 39.6 | 3.02, dd (14.0, 10.5)  2.94, dd (14.0, 4.3) | 2, 13, 14  13, 14 | 2 | 1  1 |
| 13 | 137.8 |  |  |  |  |
| 14/18 | 129.3 | 7.20, m | 12, 16 | 15/17 |  |
| 15/17 | 128.5 | 7.27, m | 13 | 14/18, 16 |  |
| 16 | 126.6 | 7.20, m | 14/18 | 15/17 |  |
| 19 | 30.5 | 1.68, m |  | 20, 21 |  |
| 20 | 16.1 | 0.28, d (6.8) | 4, 19, 21 | 19 |  |
| 21 | 18.6 | 0.65, d (6.8) | 4, 19, 20 | 19 |  |
| 22 | 168.1 |  |  |  |  |
| 23 | 135.3 |  |  |  |  |
| 24 | 114.9 | 7.27, m | 22, 26, 28 |  |  |
| 25 | 157.3 |  |  |  |  |
| 26 | 118.7 | 6.94, dd (8.1, 2.4) | 24, 25, 28 | 27 |  |
| 27 | 129.5 | 7.27, m | 23, 25 | 26, 28 |  |
| 28 | 118.8 | 7.33, m | 22, 24, 26 | 27 |  |
| 29 | 15.7 | 1.19, d (6.4) | 6, 7 | 7 |  |
| 30 | 35.6 | 1.82, qd (8.0, 3.4) | 9 | 9, 31, 32 |  |
| 31 | 14.1 | 0.84, d (8.0) | 9, 30, 32 | 30 | 34 |
| 32 | 24.1 | 1.47, ddt (13.7, 7.4, 3.4)  1.14, m | 33 | 30, 33  33 |  |
| 33 | 10.3 | 0.82, t (7.4) | 30, 32 | 32a, 32b |  |
| 34 | 22.1 | 1.25, s | 1, 10, 11, 35 |  | 1 |
| 35 | 26.5 | 1.29, s | 1, 10, 11, 34 |  | 1, 2 |
| 1-OH |  | 4.38, d (10.5) | 1, 2, 11 | 1 |  |
| 6-NH |  | 8.64, d (9.1) | 6, 22 | 6 | 7, 24, 27, 29 |
| 25-OH |  | 9.79, s |  |  |  |

| **Table S6**. ^1^H (600 MHz) and ^13^C (151 MHz) NMR data for **3** in DMSO-*d*_6_. | | | | | |
| --- | --- | --- | --- | --- | --- |
| **No.** | ***δ*_C_** | ***δ*_H_ , mult. (*J* in Hz)** | **HMBC** | **COSY** | **NOESY** |
| 1 | 202.6 |  |  |  |  |
| 2 | 76.4 | 5.69, dd (7.8, 5.6) | 3 | 12a, 12b |  |
| 3 | 167.8 |  |  |  |  |
| 4 | 76.0 | 5.04, d (5.2) | 5 | 19 |  |
| 5 | 168.2 |  |  |  |  |
| 6 | 55.9 | 4.94, dd (8.3, 3.1) | 5, 22 | 6-NH, 7 |  |
| 7 | 70.6 | 5.54, qd (6.4, 2.9) |  | 6, 29 | 6-NH |
| 8 | 167.2 |  |  |  |  |
| 9 | 76.7 | 4.80, d (7.3) | 8, 10, 30, 31, 32 | 30 |  |
| 10 | 171.0 |  |  |  |  |
| 11 | 54.2 |  |  |  |  |
| 12 | 37.2 | 3.15, dd (14.1, 5.6)  3.05, dd (14.1, 7.8) | 13, 14  1, 2, 13, 14 | 2 |  |
| 13 | 135.4 |  |  |  |  |
| 14/18 | 129.6 | 7.20, m | 12, 13, 16 | 15/17 |  |
| 15/17 | 128.4 | 7.29, m | 13 | 14/18, 16 |  |
| 16 | 127.0 | 7.25, m | 14/18 | 15/17 |  |
| 19 | 30.0 | 2.02, m |  | 4, 20, 21 |  |
| 20 | 16.8 | 0.68, d (6.8) | 4, 19, 21 | 19 |  |
| 21 | 17.9 | 0.82, d (6.8) | 4, 19, 20 | 19 |  |
| 22 | 167.6 |  |  |  |  |
| 23 | 135.0 |  |  |  |  |
| 24 | 114.8 | 7.29, m | 22, 26, 28 |  |  |
| 25 | 157.3 |  |  |  |  |
| 26 | 118.6 | 6.95, dd (7.9, 2.4) | 24, 25, 28 | 27 |  |
| 27 | 129.2 | 7.29, m | 23, 25 | 26, 28 |  |
| 28 | 118.4 | 7.33, m | 22, 24, 26 | 27 |  |
| 29 | 15.7 | 1.23, m | 6, 7 | 7 |  |
| 30 | 29.8 | 2.02, m |  | 9, 31, 32 |  |
| 31 | 17.7 | 0.88, d (2.6) | 9, 30, 32 | 30 |  |
| 32 | 17.6 | 0.87, d (2.6) | 9, 30, 31 | 30 |  |
| 33 | 21.2 | 1.34, s | 1, 10, 11, 34 |  |  |
| 34 | 21.3 | 1.21, s | 1, 10, 11, 33 |  |  |
| 6-NH |  | 8.64, d (8.3) | 5, 22 | 6 | 7, 29 |
| 25-OH |  | 8.42, s |  |  |  |

| **Table S7**. ^1^H (600 MHz) and ^13^C (151 MHz) NMR data for **4** in DMSO-*d*_6_. | | | | | |
| --- | --- | --- | --- | --- | --- |
| **No.** | ***δ*_C_** | ***δ*_H_ , mult. (*J* in Hz)** | **HMBC** | **COSY** | **NOESY** |
| 1 | 77.9 | 3.29, m | 10 | 1-OH |  |
| 2 | 71.8 | 5.38, dd (10.4, 4.4) | 3 | 12a, 12b | 14/18 |
| 3 | 168.0 |  |  |  |  |
| 4 | 75.5 | 5.28, d (3.4) | 3, 5, 19, 20, 21 | 19 |  |
| 5 | 168.1 |  |  |  |  |
| 6 | 55.5 | 5.04, dd (9.0, 3.4) | 5 | 6-NH | 29 |
| 7 | 70.9 | 5.50, qd (6.4, 3.4) |  | 29 |  |
| 8 | 167.8 |  |  |  |  |
| 9 | 75.7 | 4.50, d (7.9) | 8, 10, 30, 31, 32 | 30 |  |
| 10 | 174.9 |  |  |  |  |
| 11 | 45.4 |  |  |  |  |
| 12 | 39.2 | 3.04, dd (14.0, 10.4)  2.95, dd (14.0, 4.4) | 1, 2, 13, 14  2, 13, 14 | 2 |  |
| 13 | 137.7 |  |  |  |  |
| 14/18 | 129.1 | 7.21, m | 12, 16 | 15/17 | 2 |
| 15/17 | 128.3 | 7.27, m | 13 | 14/18, 16 | 1 |
| 16 | 126.4 | 7.21, m | 14/18 | 15/17 |  |
| 19 | 30.4 | 1.70, m |  | 20, 21 | 24 |
| 20 | 16.0 | 0.30, d (6.8) | 4, 19, 21 | 19 |  |
| 21 | 18.5 | 0.67, d (6.8) | 4, 19, 20 | 19 |  |
| 22 | 167.9 |  |  |  |  |
| 23 | 135.2 |  |  |  |  |
| 24 | 114.8 | 7.27, m | 22, 25, 26, 28 |  |  |
| 25 | 157.3 |  |  |  |  |
| 26 | 118.5 | 6.94, dd (8.1, 2.5) | 24, 25, 28 | 27 |  |
| 27 | 129.2 | 7.27, m | 23, 25 | 26, 28 |  |
| 28 | 118.8 | 7.34, m | 22, 24, 26 | 27 |  |
| 29 | 15.5 | 1.20, d (6.4) | 6, 7 | 7 |  |
| 30 | 29.6 | 1.99, m |  | 9, 31, 32 |  |
| 31 | 18.1 | 0.89, d (6.9) | 9, 30, 32 | 30 |  |
| 32 | 17.7 | 0.91, d (6.6) | 9, 30, 31 | 30 |  |
| 33 | 22.0 | 1.25, s | 1, 10, 11, 34 |  |  |
| 34 | 26.5 | 1.29, s | 1, 10, 11, 33 |  |  |
| 1-OH |  | 4.38, m |  | 1 |  |
| 6-NH |  | 8.62, d (9.1) | 5, 22 | 6 | 29 |
| 25-OH |  | 8.42, s |  |  |  |

| **Table S8**. The deduced orfs of neoantimycin biosynthetic gene cluster. | | | |
| --- | --- | --- | --- |
| **Genes** | **Proposed functions** | **Genbank No.** | |
| ***nat*A** | RNA polymerase sigma factor | AWX24481.1 | |
| ***nat*B** | NRPS (C-A-T, C-A-KR-T, C-A-KR-T) | AWX24482.1 | |
| ***nat*C** | PKS (KS-AT-MT-T) | AWX24483.1 | |
| ***nat*D** | NRPS (C-A-KR-T-TE) | AWX24484.1 | |
| ***nat*E** | NADPH dependent ketoreductase | AWX24485.1 | |
| ***nat*R** | Type II thioesterase | AWX24486.1 |  |
| ***nat*P** | phosphopantetheinyl transferase | AWX24487.1 | |
| ***nat*F** | acyl-CoA ligase | AWX24488.1 | |
| ***nat*G** | acyl carrier protein | AWX24489.1 | |
| ***nat*H** | phenylacetate-CoA oxygenase | AWX24490.1 | |
| ***nat*I** | phenylacetate-CoA oxygenase subunit | AWX24491.1 | |
| ***nat*J** | phenylacetate-CoA oxygenase, PaaI subunit | AWX24492.1 | |
| ***nat*K** | phenylacetate-CoA oxygenase, PaaJ subunit | AWX24493.1 | |
| ***nat*L** | phenylacetate-CoA oxygenase/reductase PaaK subunit | AWX24494.1 | |
| ***nat*N** | tryptophan 2,3-dioxygenase | AWX24495.1 | |
| ***nat*O** | lipase/esterase | AWX24496.1 | |
| ***nat*Q** | kynureninase | AWX24497.1 | |

| **Table S9.** The Cytotoxic Activities (IC_50_, *μ*M) of **1-4** Against Six Other Human Cancer Cell Lines | | | | | | |
| --- | --- | --- | --- | --- | --- | --- |
|  | **A549 *^a^*** | **HT29 *^b^*** | **HCT-8 *^c^*** | **SGC7901 *^d^*** | **HepG2 *^e^*** | **Hela *^f^*** |
| **1** | 7.70 ± 0.07 | 19.4 ± 0.1 | 17.9 ± 1.4 | 11.9 ± 0.1 | 13.4 ± 0.1 | 18.2 ± 0.2 |
| **2** | 2.99 ± 0.02 | 14.9 ± 0.3 | 9.23 ± 0.16 | 9.57 ± 0.22 | 15.2 ± 0.7 | 15.3 ± 1.5 |
| **3** | 2.32 ± 0.01 | 17.6 ± 0.9 | 10.9 ± 0.8 | 6.63 ± 0.17 | 17.5 ± 0.6 | 23.0 ± 0.4 |
| **4** | 2.97 ± 0.01 | 17.2 ± 0.8 | 8.95 ± 0.13 | 10.1 ± 0.3 | 18.3 ± 0.7 | 23.6 ± 0.5 |
| Cisplatin | 10.4 ± 0.1 | 19.7 ± 0.2 | 11.2 ± 1.1 | 2.31 ± 0.10 | 3.07 ± 0.04 | 3.03 ± 0.06 |
| Human cancer cells: ***^a^*** lung cancer cells; ***^b^*** colorectal cancer cells; ***^c^*** ileocecal cancer cells; ***^d^*** gastric carcinoma cells;  ***^e^*** liver carcinoma cells; ***^f^*** cervical cancer cells. Values shown are means ± SD of triplicate determinations. | | | | | | |

**SII. Structure Elucidation of Compounds 1-4**

The ^1^H-NMR spectrum of compound **1** exhibited seven methyl groups and five oxygenated or nitrogen-bearing methines and two exchangeable proton signals from NH and OH. The ^13^C and DEPT NMR spectra showed 31 carbon signals, among which *δ*_C_ 128.6 and 118.5 had double intensity and *δ*_C_ 129.2 had threefold intensity. The downfield zone of ^13^C-NMR exhibited characteristic resonances in NATs for one ketone (*δ*_C_ 202.5) and five carbonyl groups of ester or acylamino (*δ*_C_ 167-171). The direct ^1^H-^13^C connections were confirmed by HSQC and the tabulated NMR spectral data for **1** is shown in Table S4. The two pendant moieties, benzyl and 3-HBA, were correlated to the nine aromatic proton resonances at *δ*_H_ 7.33 (3H, m), 7.27 (4H, m), 7.25 (1H, m) and 6.94 (1H, ddd, *J*= 8.0, 2.5, 1.0 Hz), of which the five protons at *δ*_H_ 7.27 (2H, H-14/18), 7.33 (2H, H-15/17), 7.25 (1H, H16) constructed a monosubstituted benzene. The remaining four aromatic proton signals with ^1^H-^1^H correlations from H26 (*δ*_H_ 6.94) to H28 (*δ*_H_ 7.33) through H27 (*δ*_H_ 7.27), together with ^1^H-^13^C long-ranged correlations from 25-OH (*δ*_H_ 9.69) to C24 (*δ*_C_ 114.8), C25 (*δ*_C_ 157.2) and C26 (*δ*_C_ 118.5), from H27 to C23 (*δ*_C_ 135.2), and from H24 (*δ*_H_ 7.27) and H28 to C22 (*δ*_C_ 167.9) suggested the attachment of 3-HBA moiety at C6 position. The downfield chemical shift value (*δ*_C_ 157.2) for C25 indicated a hydroxyl group attached to the carbon, confirming the presence of 3-HBA moiety in **1**.

The major differences between **1** and **2** are the presence of an extra exchangeable proton signal (*δ*_H_ 4.38) in ^1^H NMR and the absence of ketone carbonyl signal in ^13^C NMR (Table S4 and S5), which is indicative of a substitution of hydroxy group at C1 in **2** instead of the ketone in **1**. This proposal was corroborated by the COSY correlation between 1-OH (*δ*_H_ 4.38) and H1 (*δ*_H_ 3.28) and HMBC correlations from 1-OH to C1 (*δ*_C_ 78.2), C2 (*δ*_C_ 72.0) and C11(*δ*_C_ 45.5), respectively (Table S5 and Fig. S1).

The ^1^H and ^13^C NMR data of **3** (Table S6) were almost identical to **1**, except the absence of a methylene signal (*δ*_C_ 24.1, C-32) of **1**. The structure of C9-isopropyl in **3**, rather than C9-isobutyl in **1**, was elucidated by ^1^H-^1^H correlations between two methyl groups H31/ 32 (*δ*_H_ 0.88 and 0.87) and the methine H30 (*δ*_H_ 2.02) (Table S6 and Fig. S1). Thus, the planer structure of **3** was determined as shown in Fig. S1.

Similar to **2**, the compound **4** also shows an extra exchangable proton signal (*δ*_H_ 4.38) in ^1^H NMR and the absence of ketone carbonyl signal in ^13^C NMR (Table S7), which suggests **4** possesses a hydroxy group at C1. ^1^H-^1^H correlations between two methyl groups H31/32 (*δ*_H_ 0.89 and 0.91) and the methine H30 (*δ*_H_ 1.99) suggest C9-isopropyl in **4**, just like **3**. And the planer structure of **4** was determined as shown in Fig. S1.

The structure elucidations of **1-4** are also supported by HR-MS/MS analysis (Table S3, Fig. S2).

**SIII. Figs. S1-S14**

**Fig. S1.** Diagnostic 2D NMR (DMSO-*d_6_*) correlations for compounds **1**-**4**.

**Fig. S2**. Identification of compounds **1**-**4** by HR-MS/MS analysis. (related to Tables S2 and S3)

1. The MS fracture spectra of compounds **1**-**4**

1. The predicted fragments of HR-MS/MS analysis


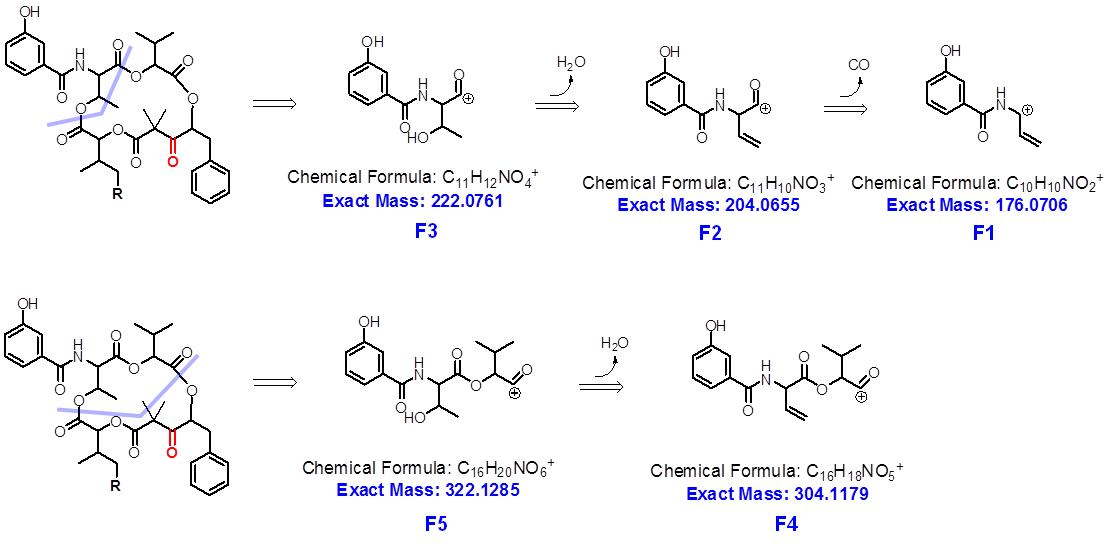


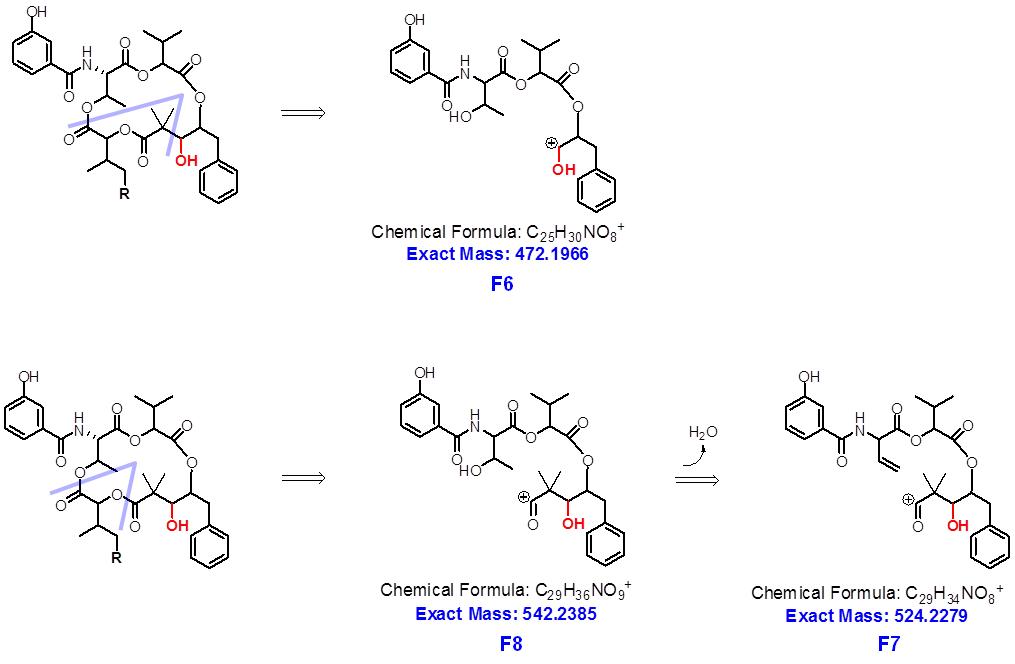


**Fig. S3**. Confirmation of the production of **1**-**4** in the fermentation extract of RJ2

**Fig. S4**. Annotation of the *nat-hyg5* gene associated type-I PKS gene cluster


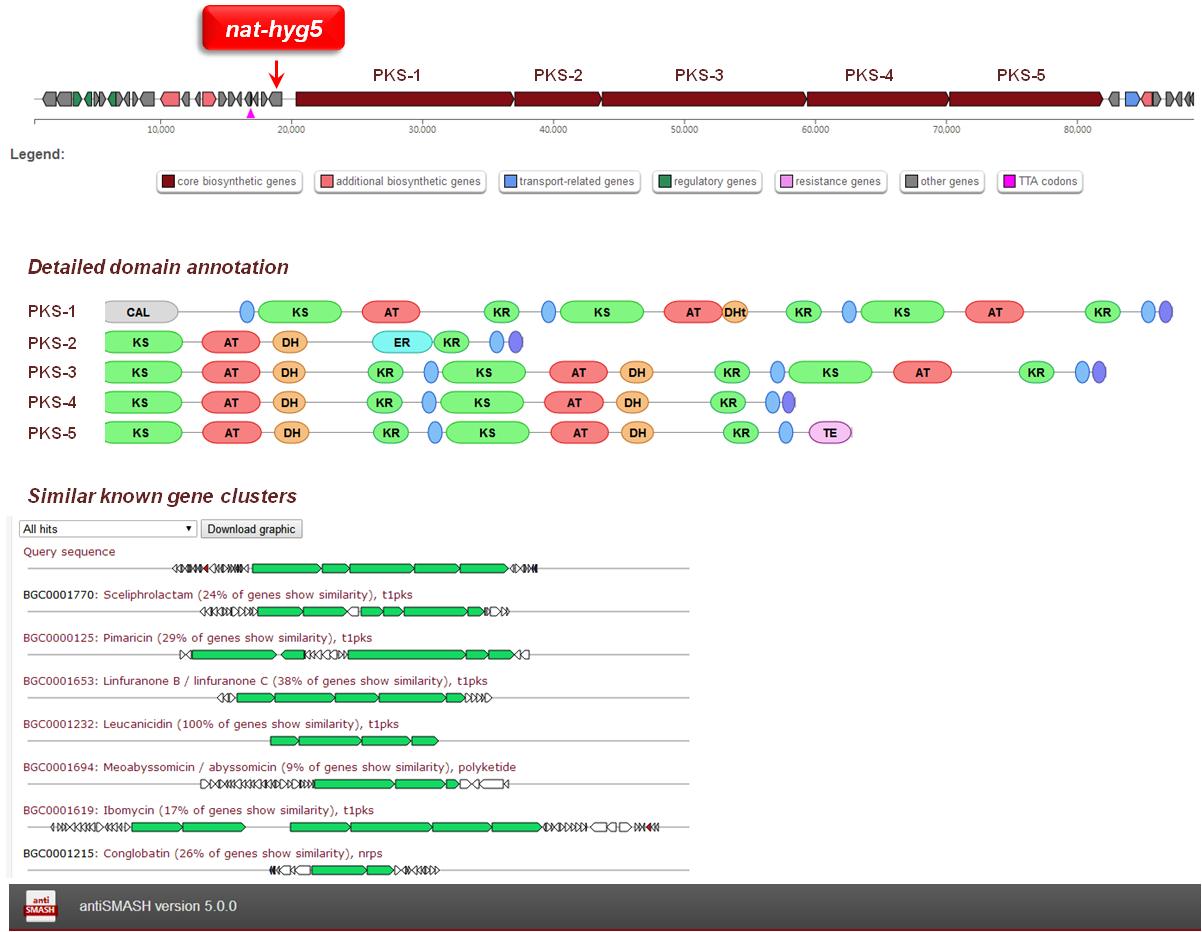


The antiSMASH 5.0 was used for the analysis (Blin K *et al*., *Nucleic Acids Res*. 2019, 47, W81-W87).

**Fig. S5**. The reaction products of the four chorismatase subfamilies

**Fig. S6**. Protein sequence alignment of the Nat-hyg5 homologues


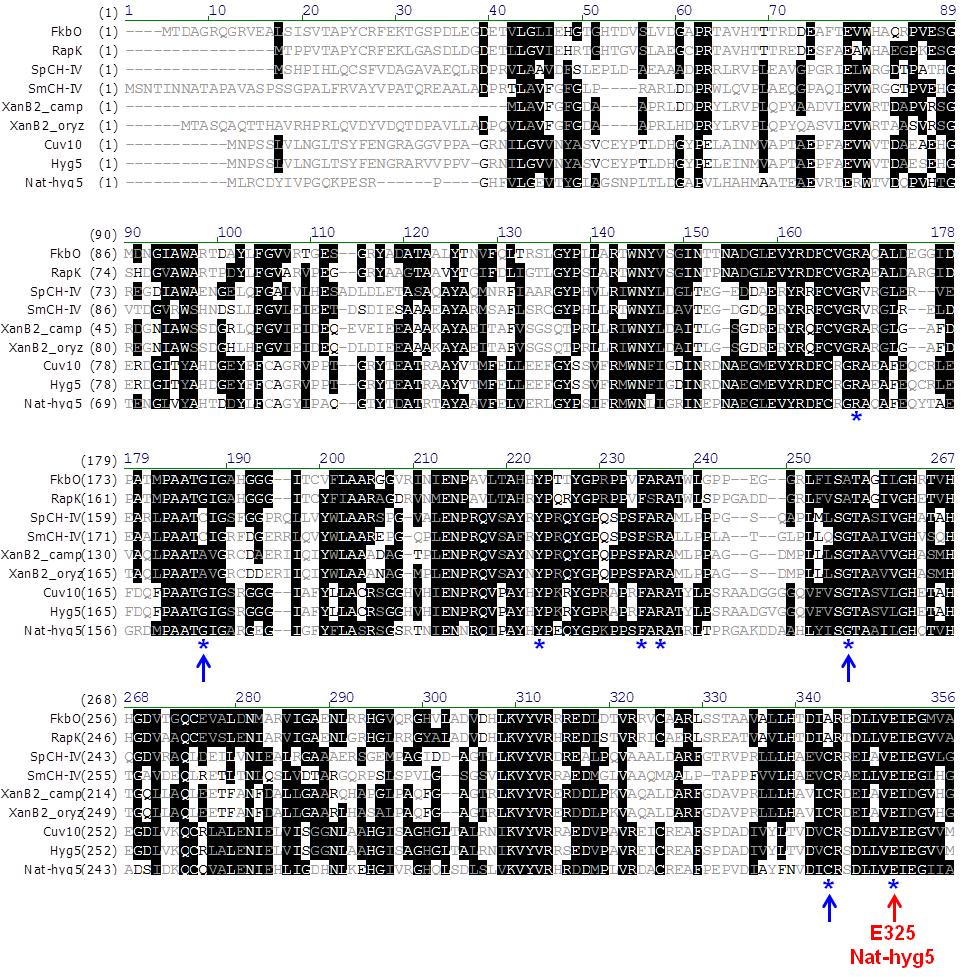


The amino acids of the active site are labeled with an asterisk (＊).The blue arrows are used to indicate the three amino acids reported to be responsible for the product selectivity in the four types of chorismatases: CH-FkbO/CH-I, CH-Hyg5/CH-II, CH-XanB2/CH-IIII, and CH-IV (Hubrich F *et al*., *J Am Chem Soc*. 2015, 137 (34), 11032-11037; Grüninger MJ *et al*., *Org. Biomol. Chem*. 2019, 17, 2092). The red arrow represents the active site selected for site mutation of *nat-hyg5* gene in the work.

The chorismatases selected for the alignment: **CH-FkbO/CH-I**: FkbO (AAF86394.1), RapK (AGP59510.1); **CH-Hyg5/CH-II**: Cuv10 (AGO98693.1), Hyg5 (AAC38060.1), Nat-hyg5 (the work, MN158725); **CH-XanB2/CH-III**: XanB2_camp (AAY51142.1), XanB2_oryz (WP_024744263.1); **CH-IV**: SmCH-IV (WP_051010542.1); SpCH-IV (KRG46027.1).

**Fig. S7.** The partial activity of NatE with compound **1** as substrate

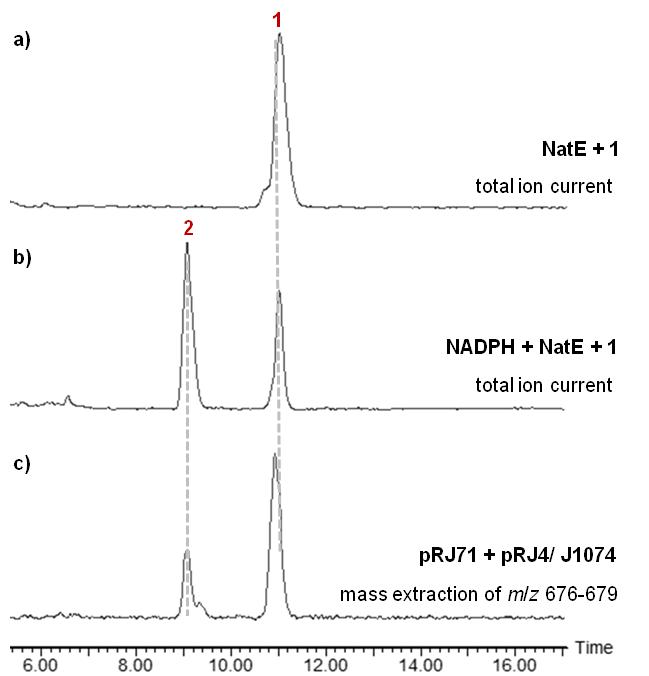


HPLC-MS analysis of the NatE enzymatic assay with the substrate of compound **1** (**a**) or with both compound **1** and the cofactor of NADPH (**b**). Compounds **1** and **2** from fermentation extract of the *S. albus* J1074 containing pRJ71and pRJ4 was used as standard in the analysis (**c**). The experiment procedures for the enzymatic assay and HPLC-MS analysis can be found in previous publication (Zhou Y *et al*., ACS Chem Biol. 2018, 13, 2153-2160).

**Fig. S8.** SDS-PAGE gel of Nat-hyg5 protein

**Fig. S9.** The Michaelis-Menten plots of Nat-hyg5-catalyzed reactions.


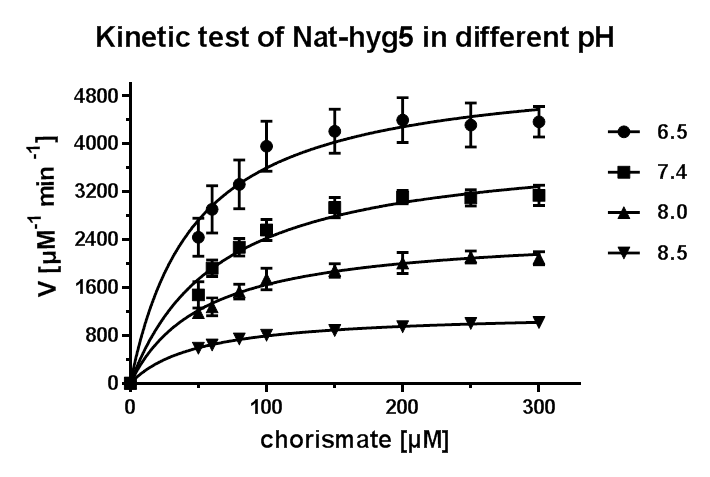


The Nat-hyg5-catalyzed reactions were carried out in pH6.8, 7.4, 8.0, and 8.5, respectively.

**Fig. S10.** Target accumulation of compounds **1** and **2** in RJ16.

(a) Graphical scheme depicts the mutant RJ16 generated from J1074 by replacing the genes *antH-L* with a cassette consisting of *nat-hyg5* gene and two strong promoters, *A35**p and *A26**p. The plasmid pRJ4 was introduced into RJ16 for targeted accumulation of compounds **1**-**4**. (b) HPLC-MS analysis of the fermentation extract of *natA-E*/RJ16 (pRJ4/RJ16) and *nat-hyg5*+*natA-E*/J1074 (pRJ71+pRJ4/RJ16) displayed with the mass extraction of *m/z* 676-679 and *m/z* 664-722, respectively.

**Fig. S11.** NMR spectra of compound **1**. (related to Table S4)

The horizontal axes in the 2D-NMRs of ***d*** *to* ***g*** and the vertical axes in the 2D-NMRs of ***d*** and ***g*** are identical to the ^1^H-NMR of ***a***. The vertical axes in the 2D-NMRs of ***e*** and ***f*** are identical to the ^13^C-NMR of ***b***.

***a.*** ^1^H-NMR spectrum of compound **1**

**
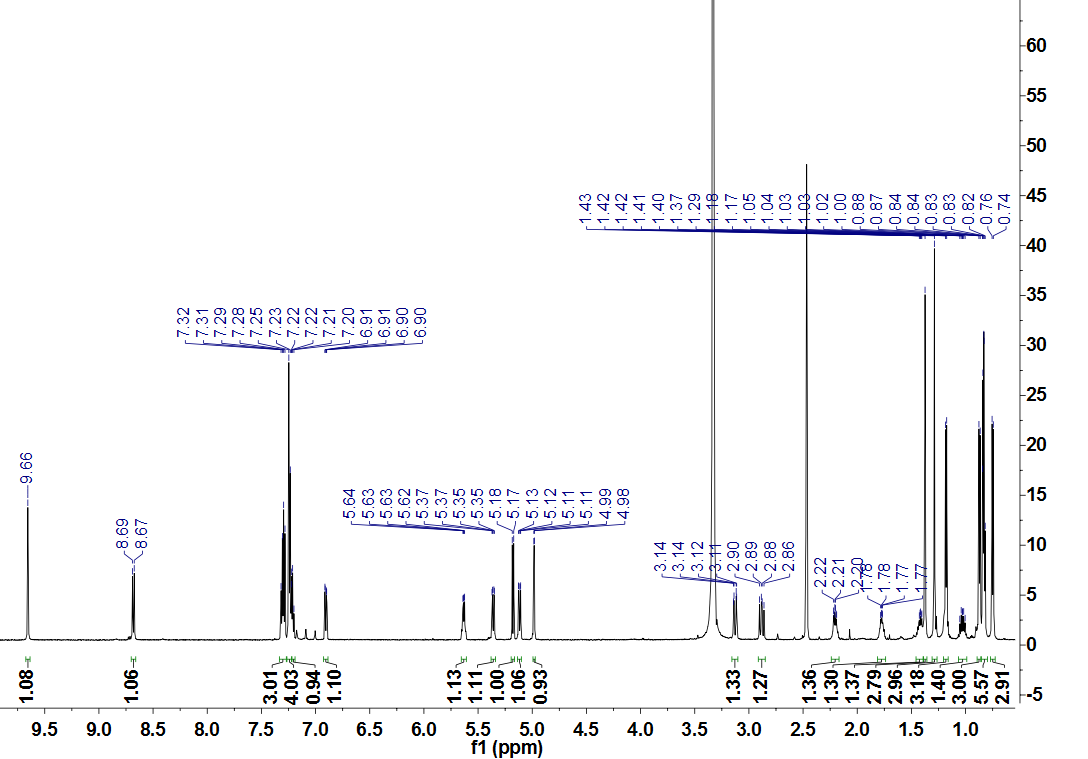
**

***b*.** ^13^C-NMR spectrum of compound **1**

**
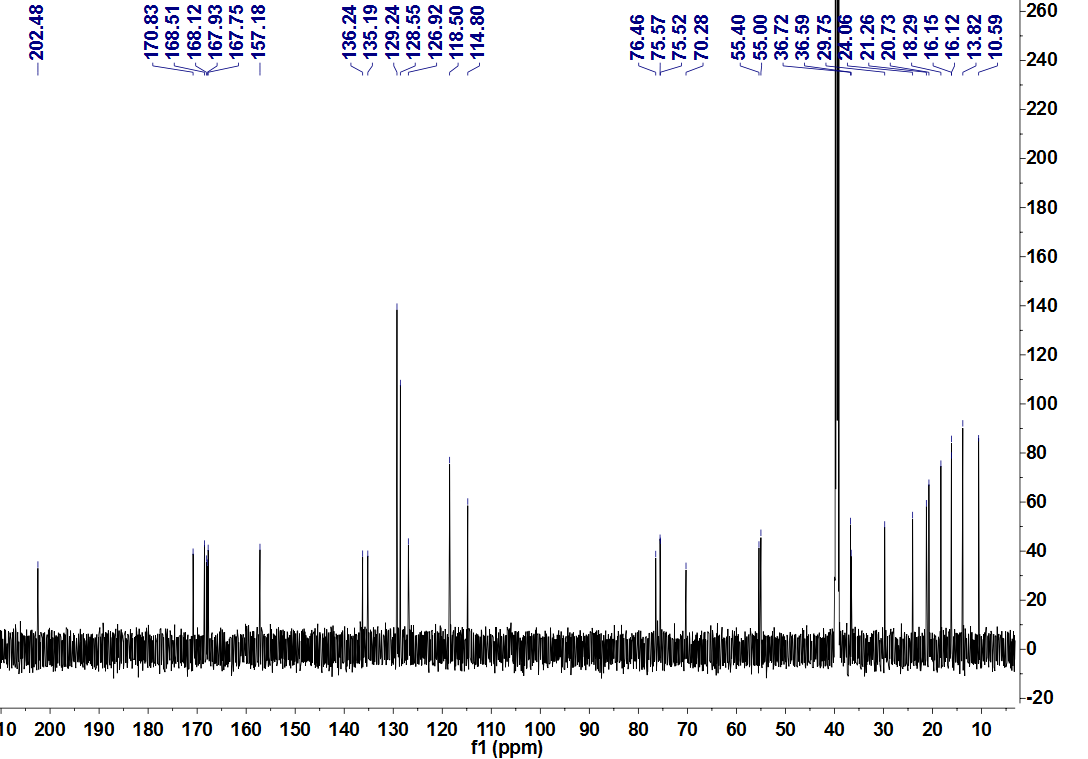
**

***c*.** DEPT 135 spectrum of compound **1**

**
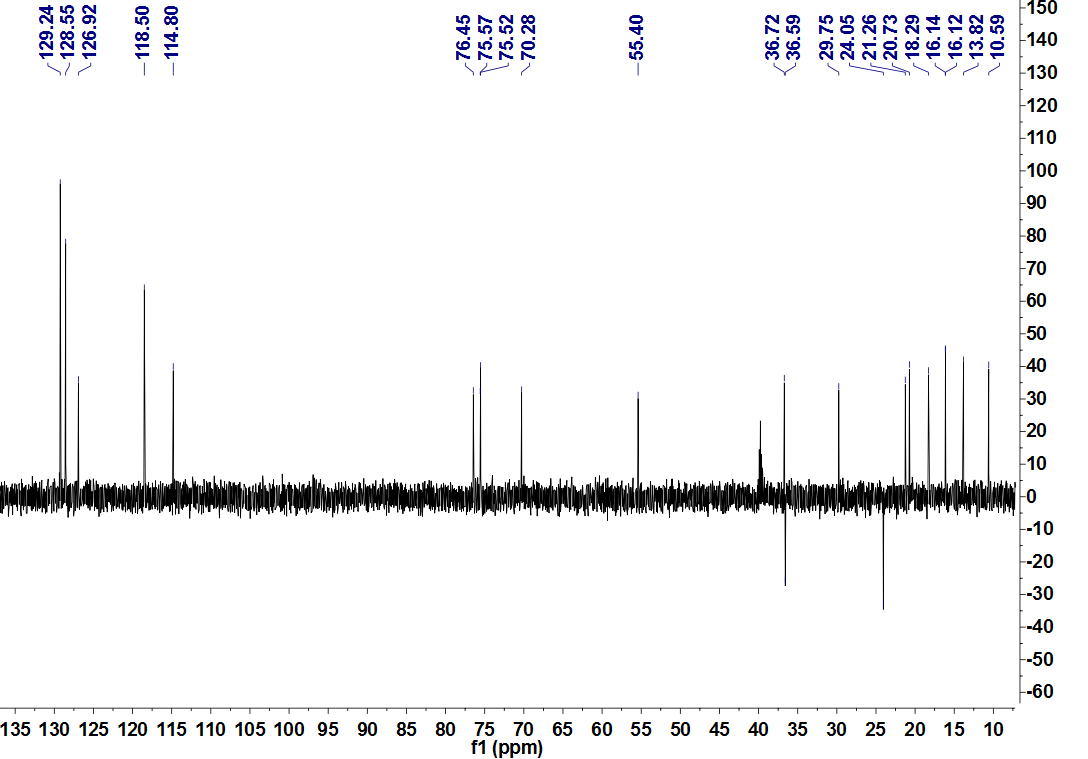
**

***d*.** ^1^H-^1^H COSY spectrum of compound **1**

**
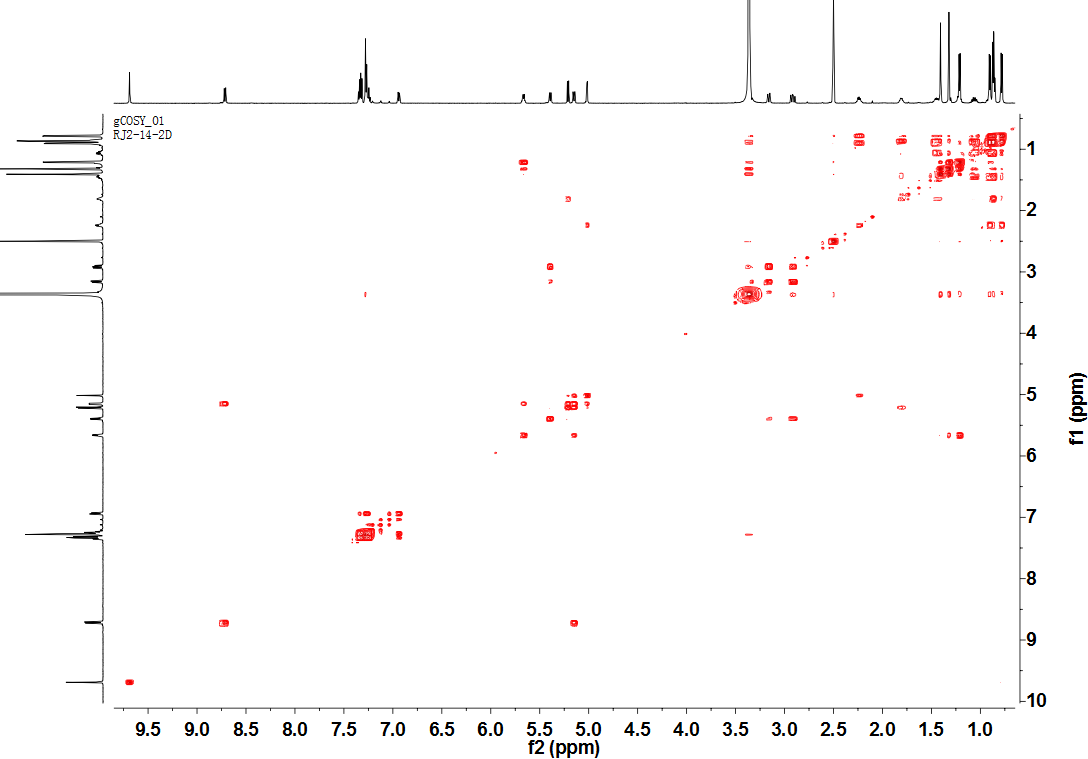
**

***e*.** HSQC spectrum of compound **1**

**
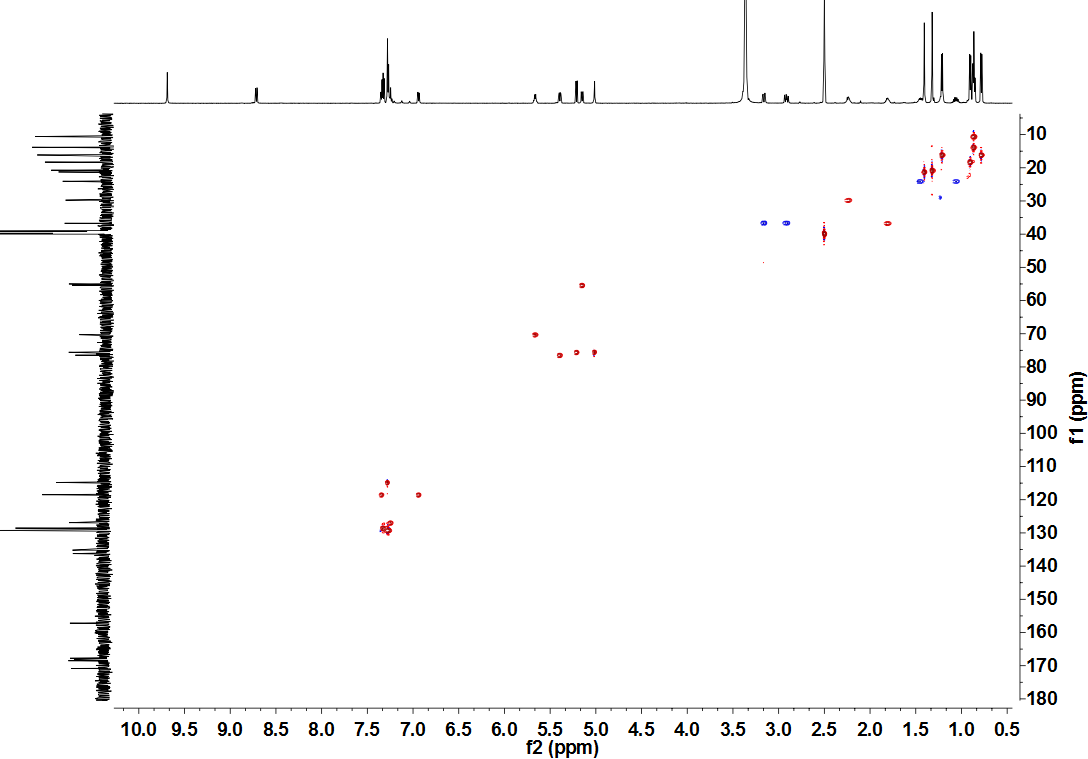
**

***f*.** HMBC spectrum of compound **1**

**
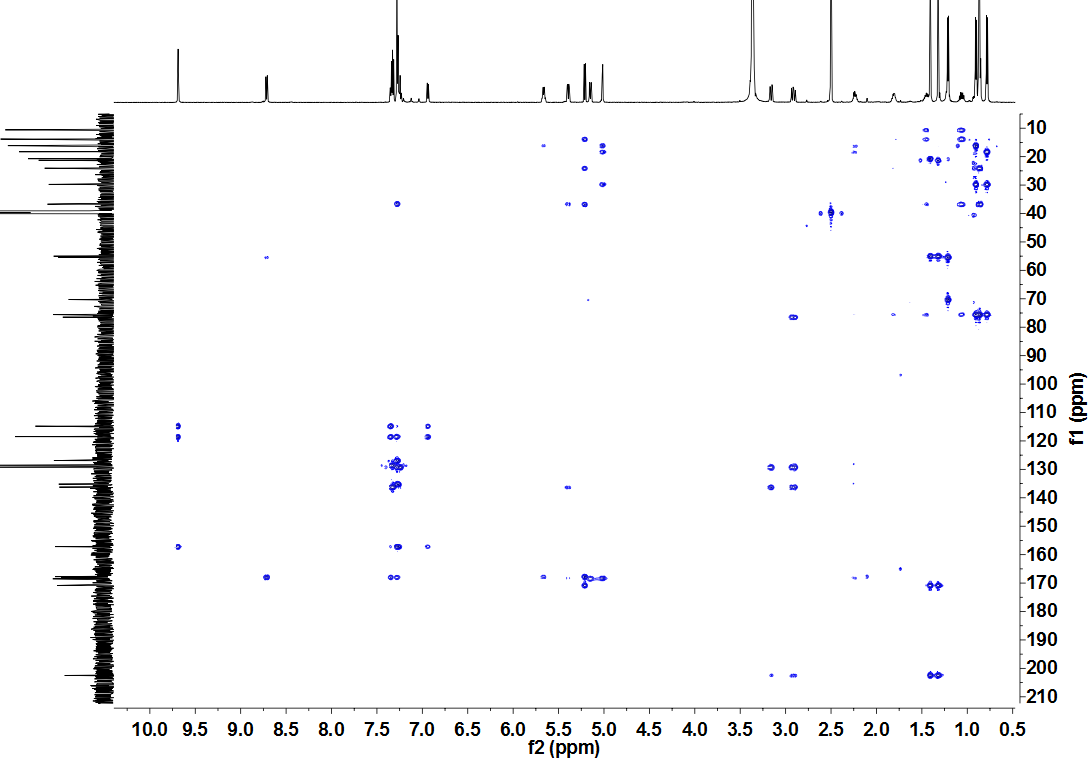
**

***g*.** ROESY spectrum of compound **1**

**
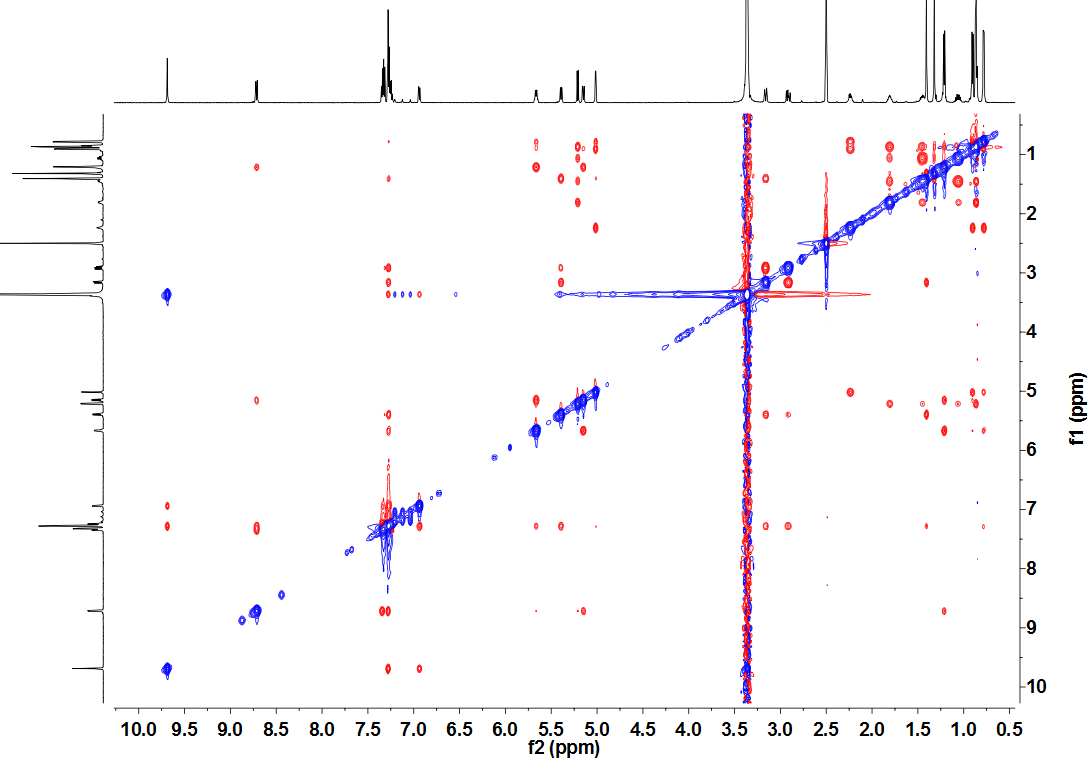
**

**Fig. S12.** NMR spectra of compound **2**. (related to Table S5)

The horizontal axes in the 2D-NMRs of ***d*** *to* ***g*** and the vertical axes in the 2D-NMRs of ***d*** and ***g*** are identical to the ^1^H-NMR of ***a***. The vertical axes in the 2D-NMRs of ***e*** and ***f*** are identical to the ^13^C-NMR of ***b***.

***a.*** ^1^H-NMR spectrum of compound **2**

**
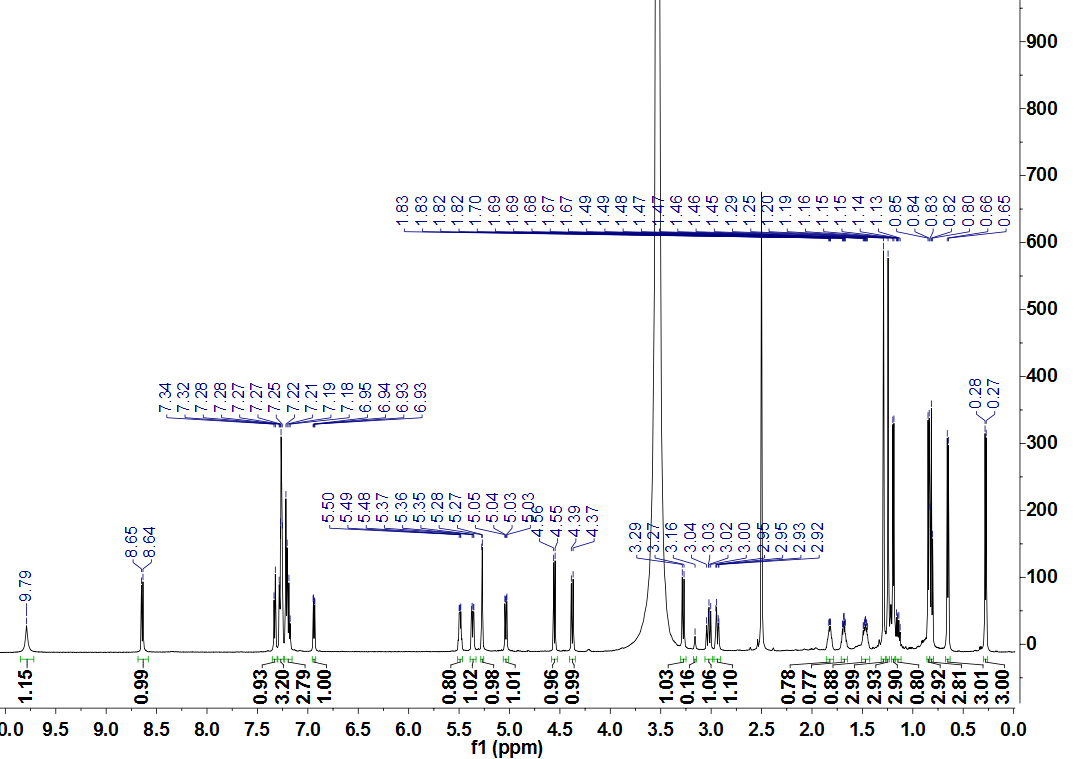
**

***b*.** ^13^C-NMR spectrum of compound **2**

**
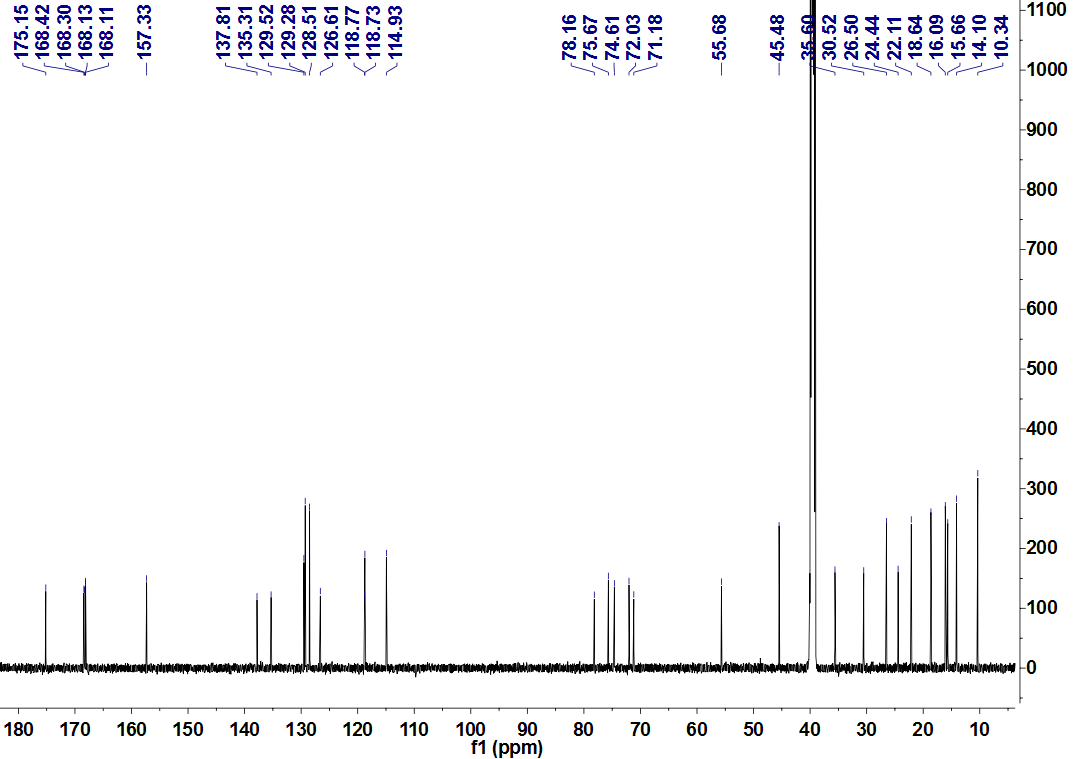
**

***c*.** DEPT 135 spectrum of compound **2**

**
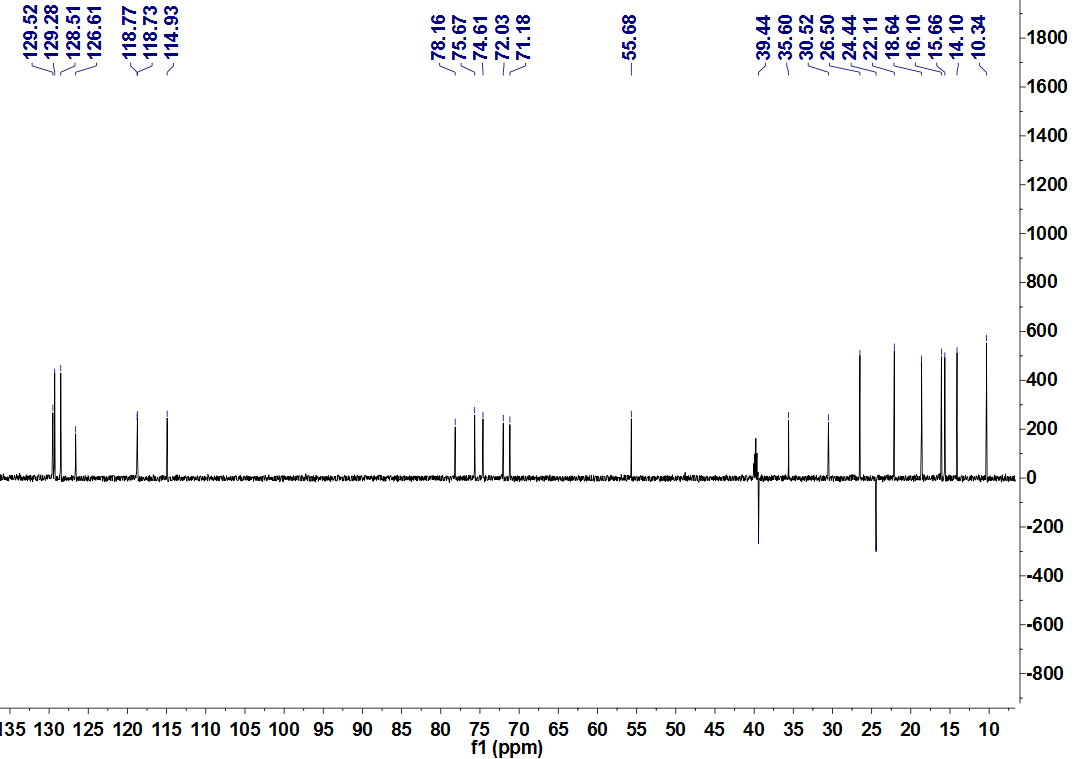
**

***d*.** ^1^H-^1^H COSY spectrum of compound **2**

**
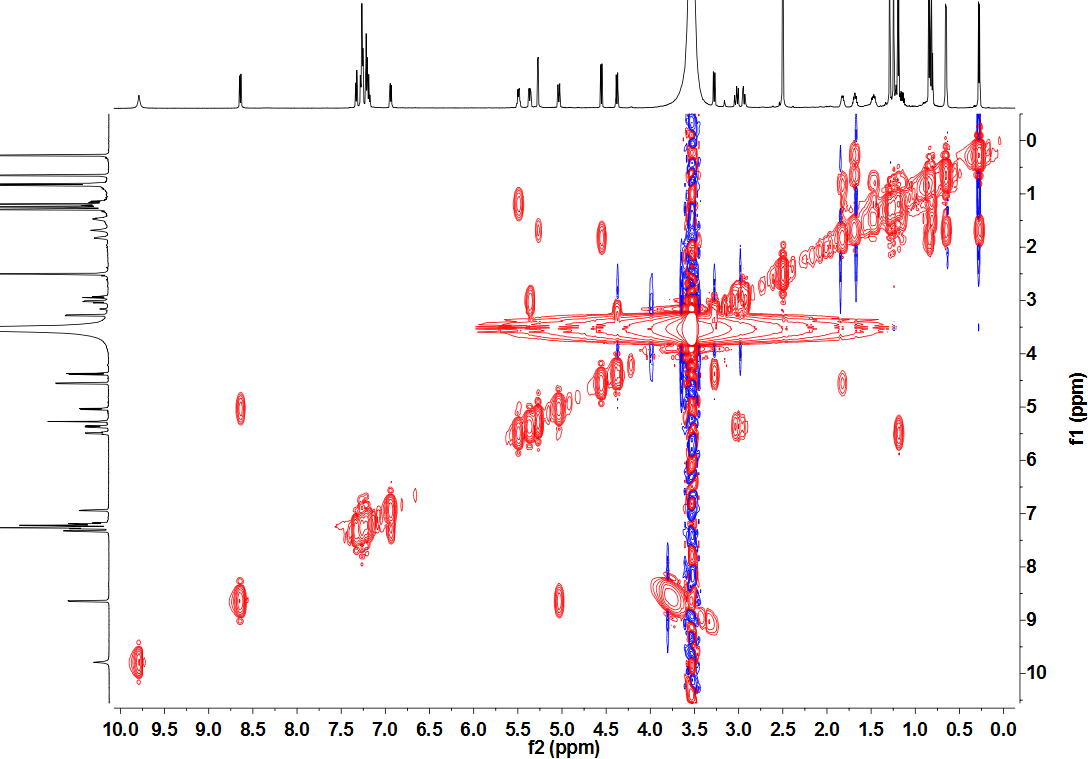
**

***e*.** HSQC spectrum of compound **2**

**
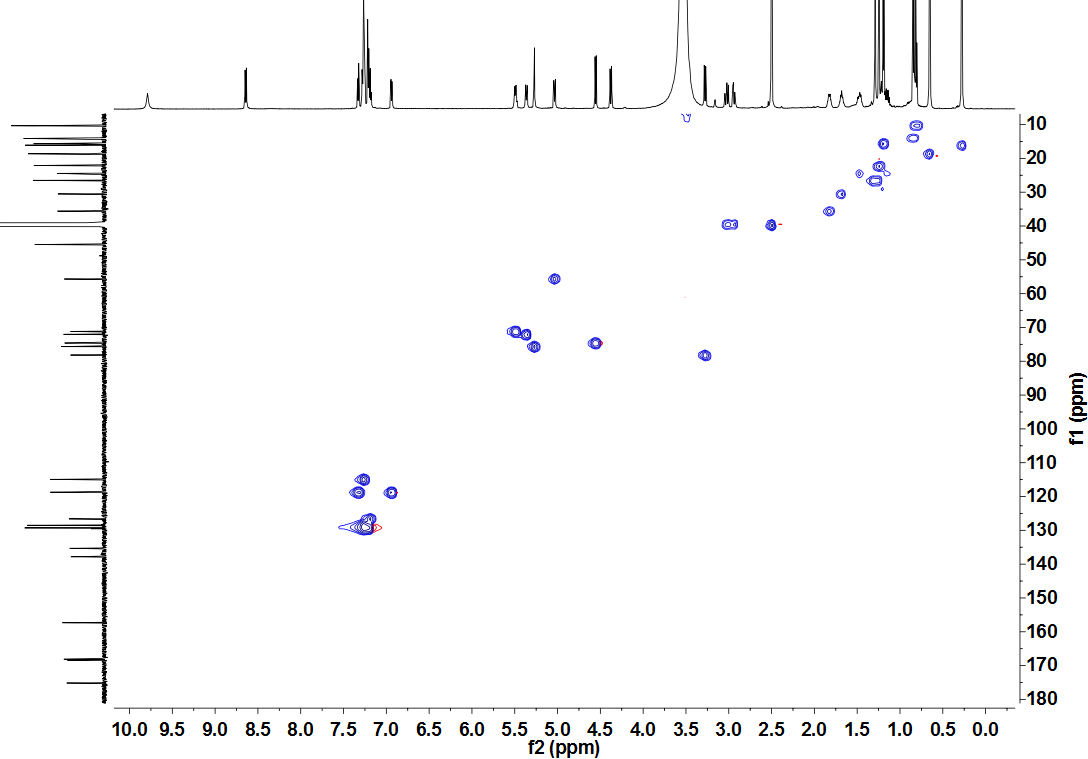
**

***f*.** HMBC spectrum of compound **2**

**
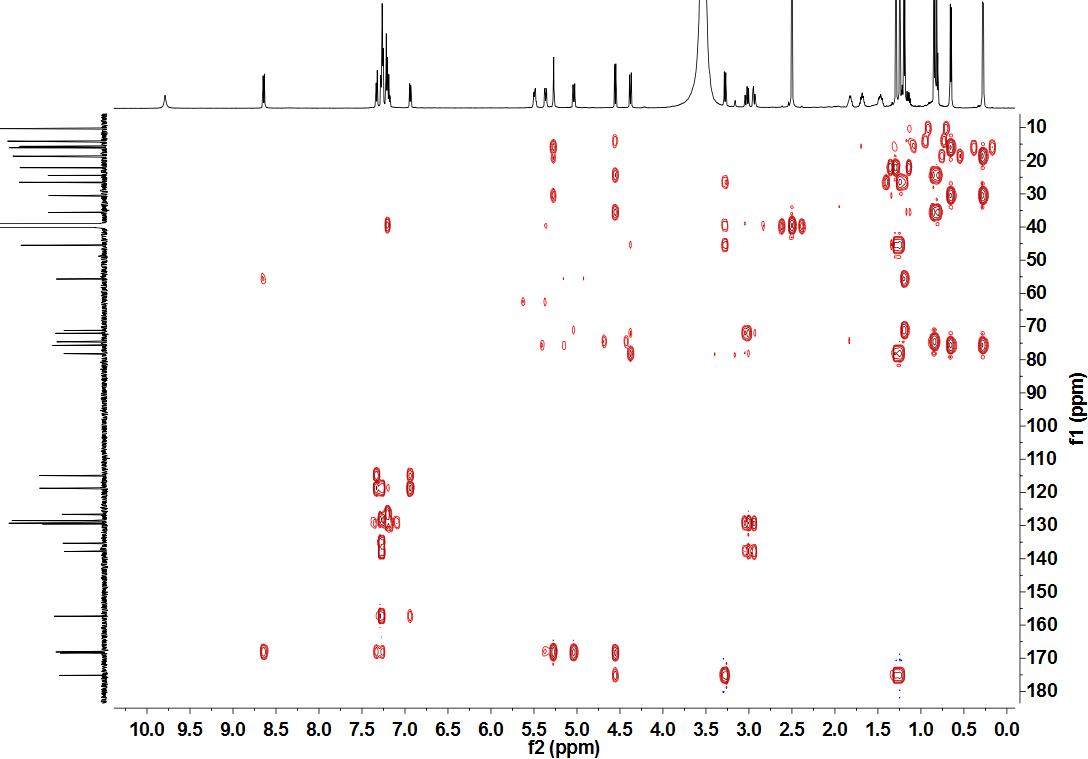
**

***g*.** NOESY spectrum of compound **2**

**
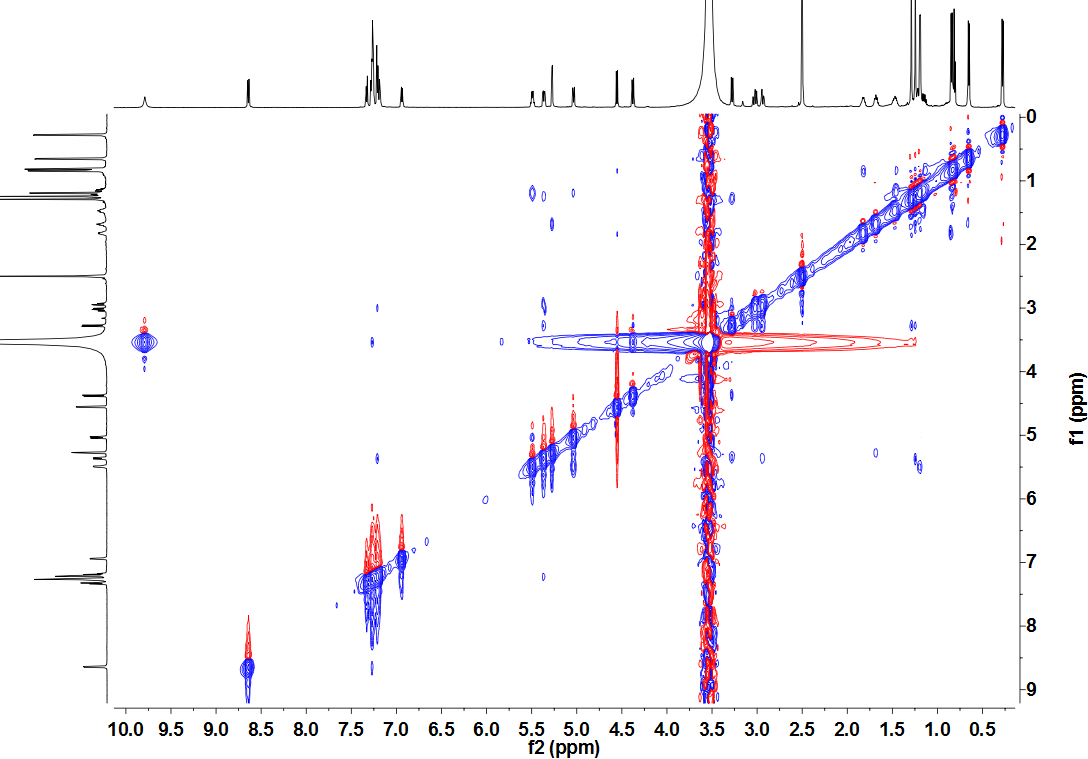
**

**Fig. S13.** NMR spectra of compound **3**. (related to Table S6)

The horizontal axes in the 2D-NMRs of ***d*** *to* ***g*** and the vertical axes in the 2D-NMRs of ***d*** and ***g*** are identical to the ^1^H-NMR of ***a***. The vertical axes in the 2D-NMRs of ***e*** and ***f*** are identical to the ^13^C-NMR of ***b***.

***a.*** ^1^H-NMR spectrum of compound **3**

**
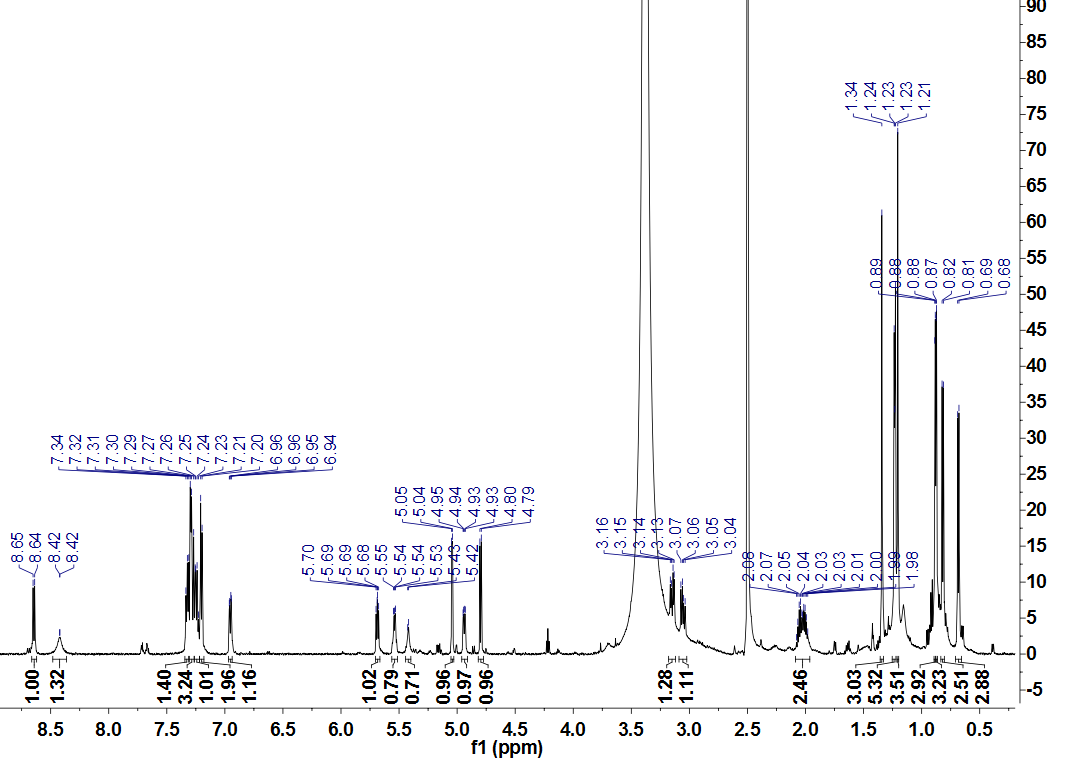
**

***b*.** ^13^C-NMR spectrum of compound **3**

**
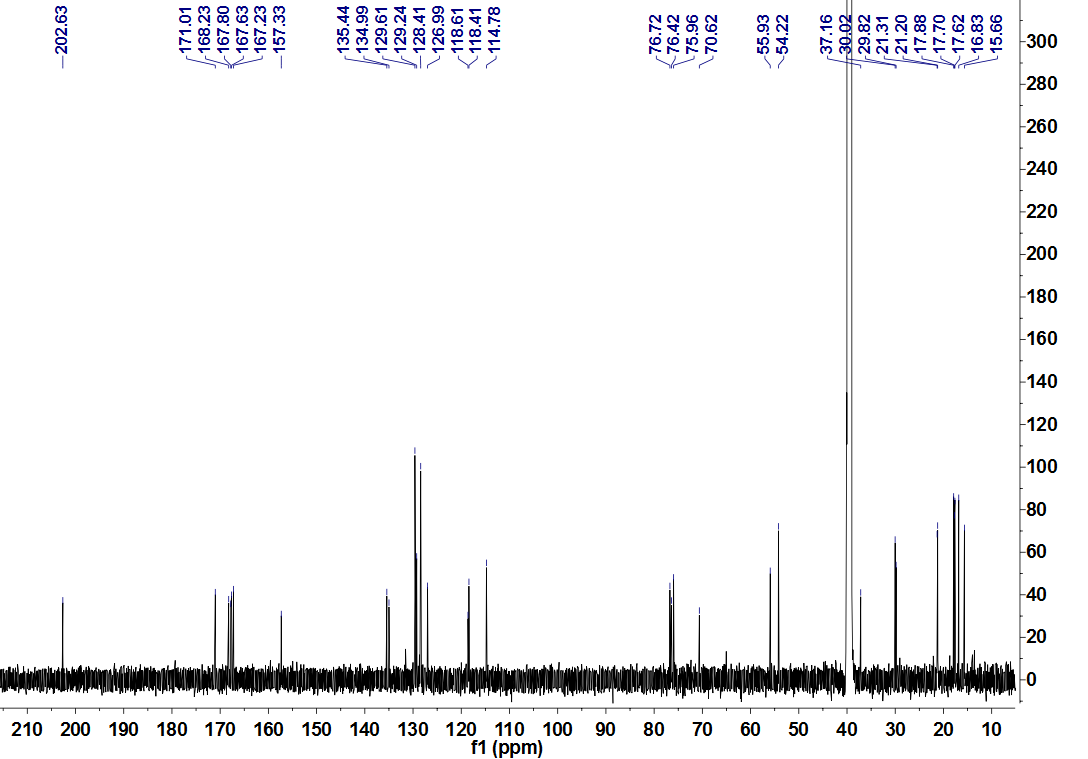
**

***c*.** DEPT 135 spectrum of compound **3**

**
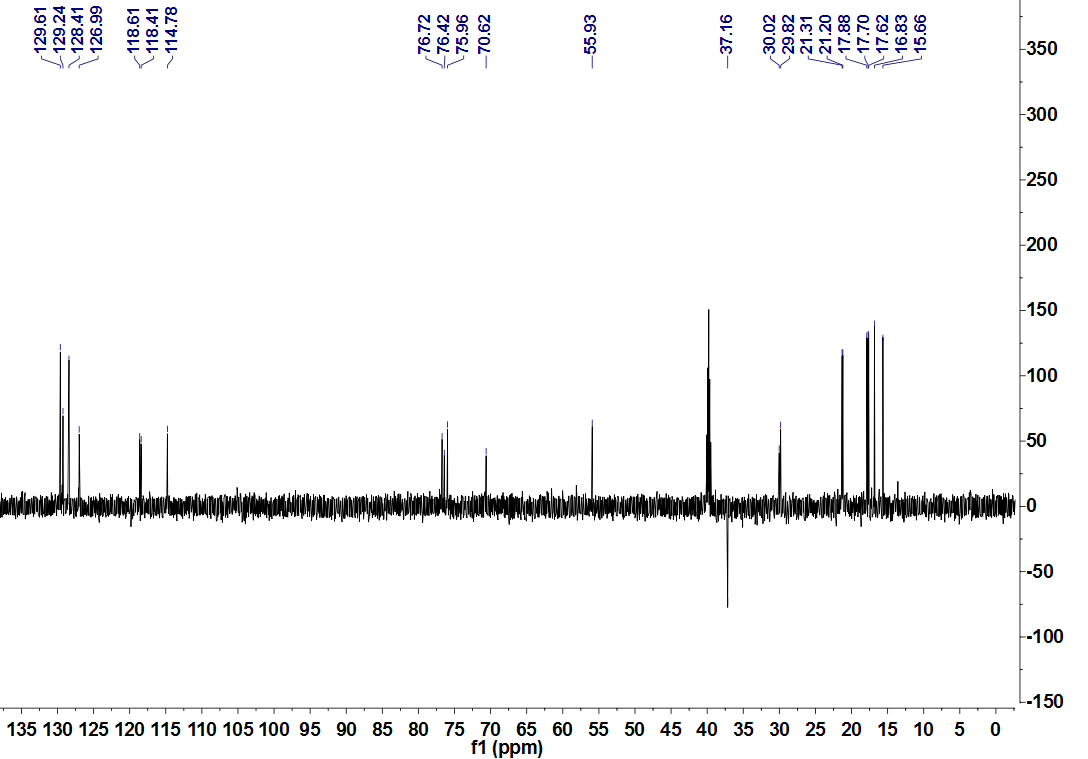
**

***d*.** ^1^H-^1^H COSY spectrum of compound **3**

**
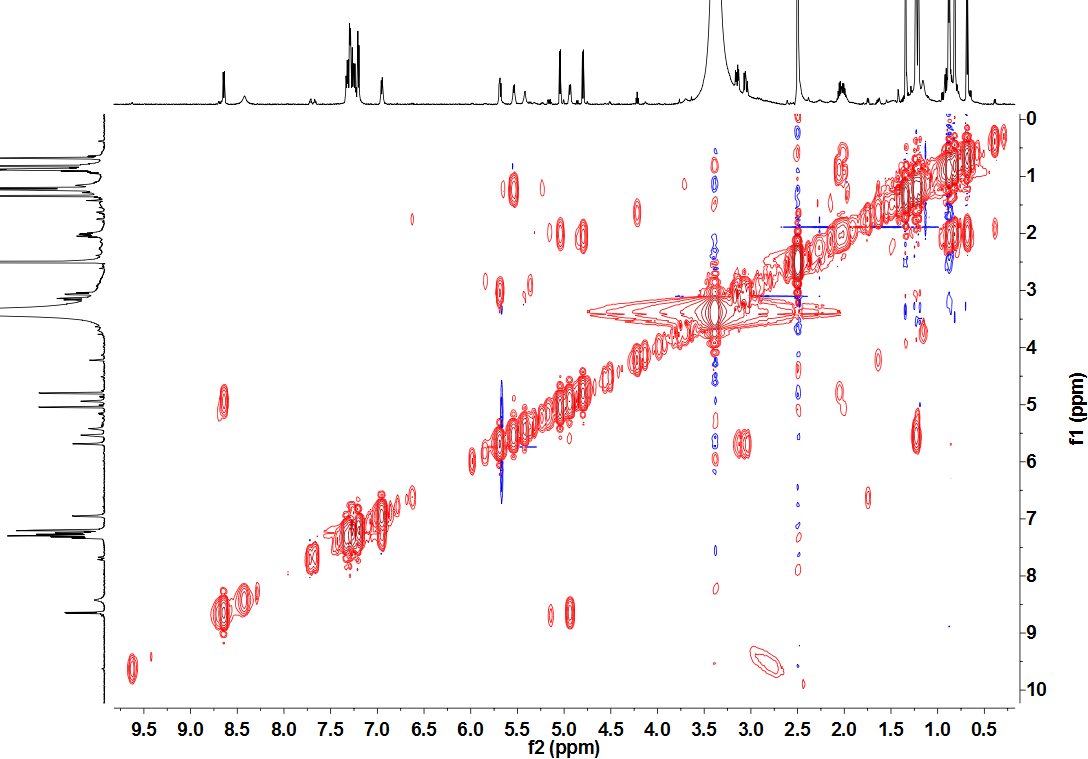
**

***e*.** HSQC spectrum of compound **3**

**
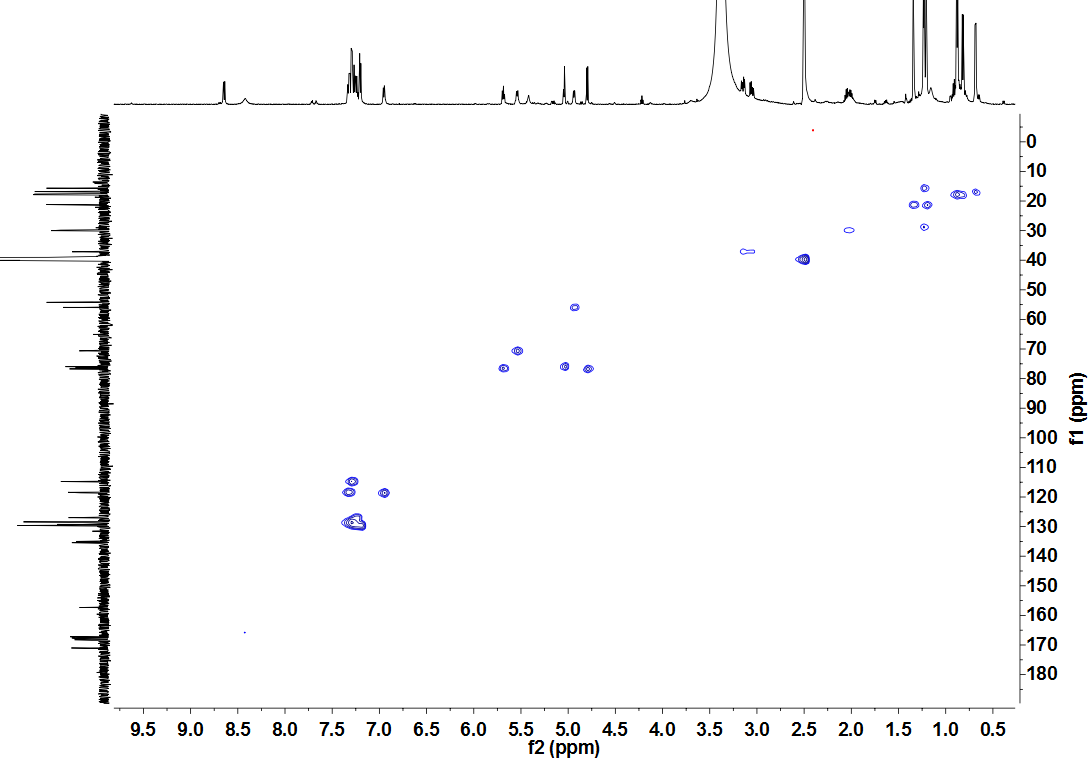
**

***f*.** HMBC spectrum of compound **3**

**
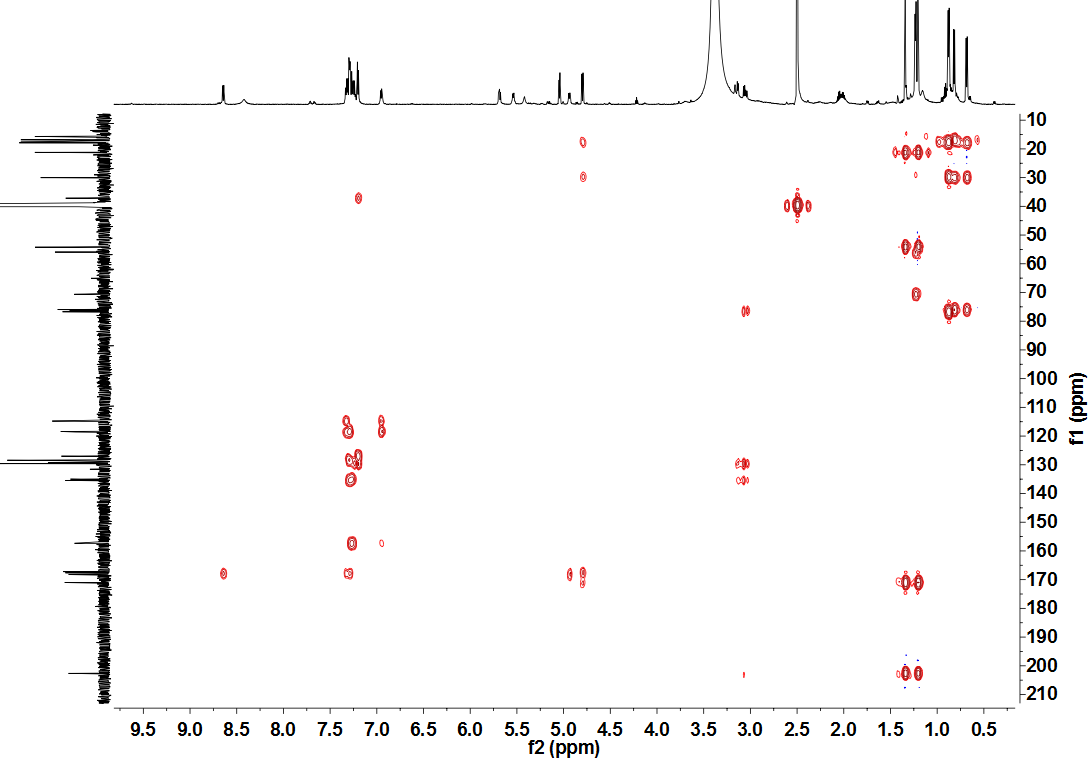
**

***g*.** NOESY spectrum of compound **3**

**
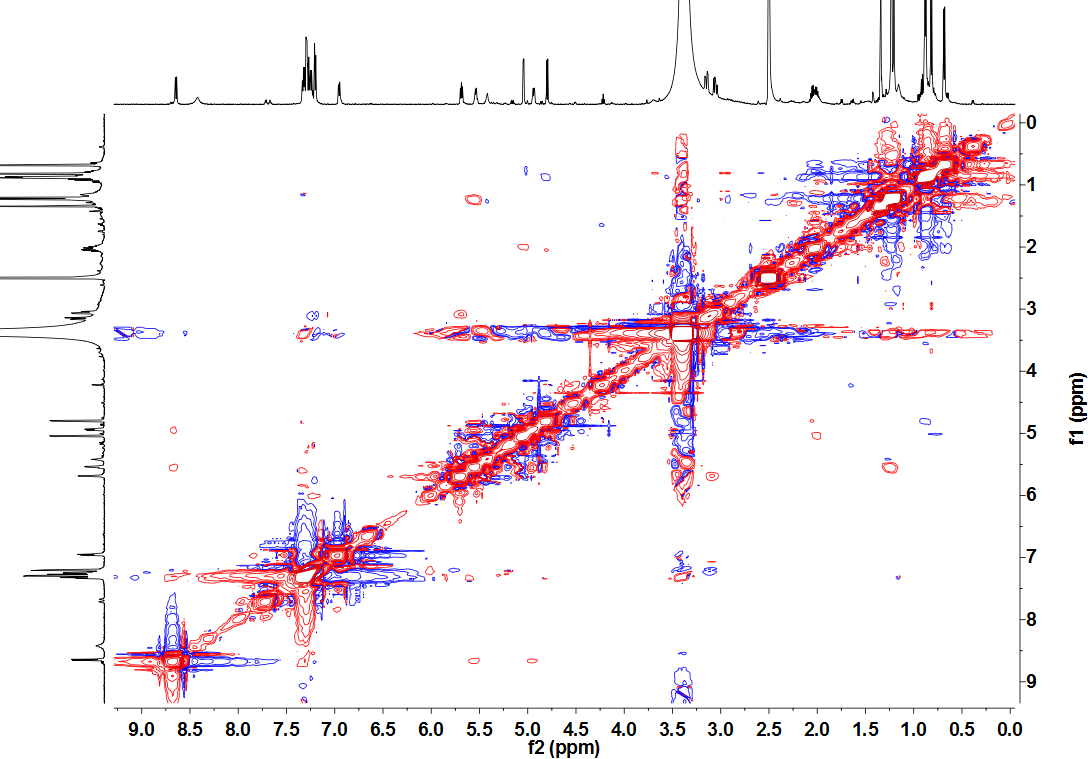
**

**Fig. S14.** NMR spectra of compound **4**. (related to Table S7)

The horizontal axes in the 2D-NMRs of ***d*** *to* ***g*** and the vertical axes in the 2D-NMRs of ***d*** and ***g*** are identical to the ^1^H-NMR of ***a***. The vertical axes in the 2D-NMRs of ***e*** and ***f*** are identical to the ^13^C-NMR of ***b***.

***a.*** ^1^H-NMR spectrum of compound **4**

**
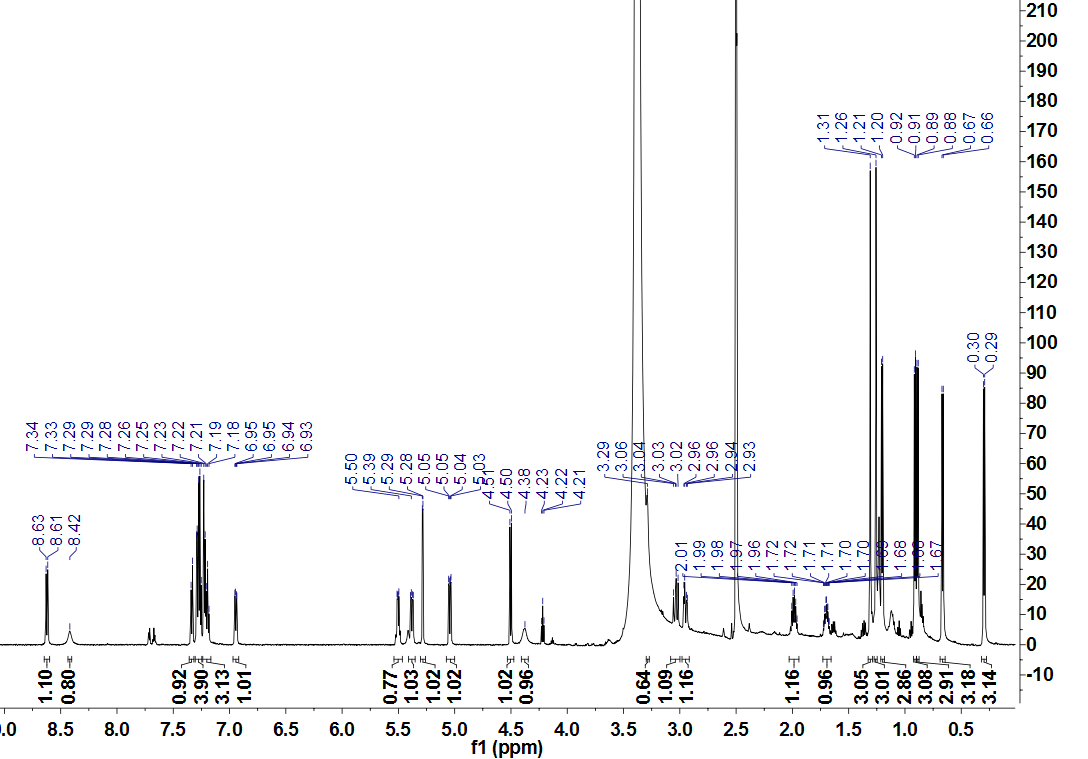
**

***b*.** ^13^C-NMR spectrum of compound **4**

**
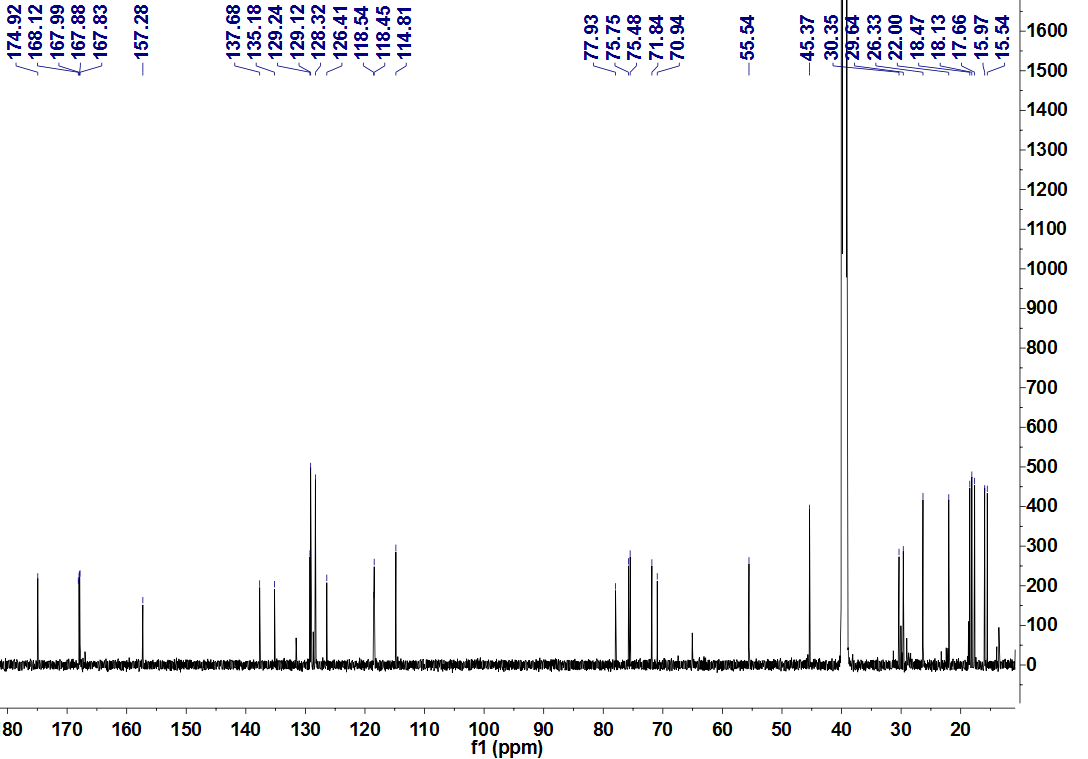
**

***c*.** DEPT 135 spectrum of compound **4**

**
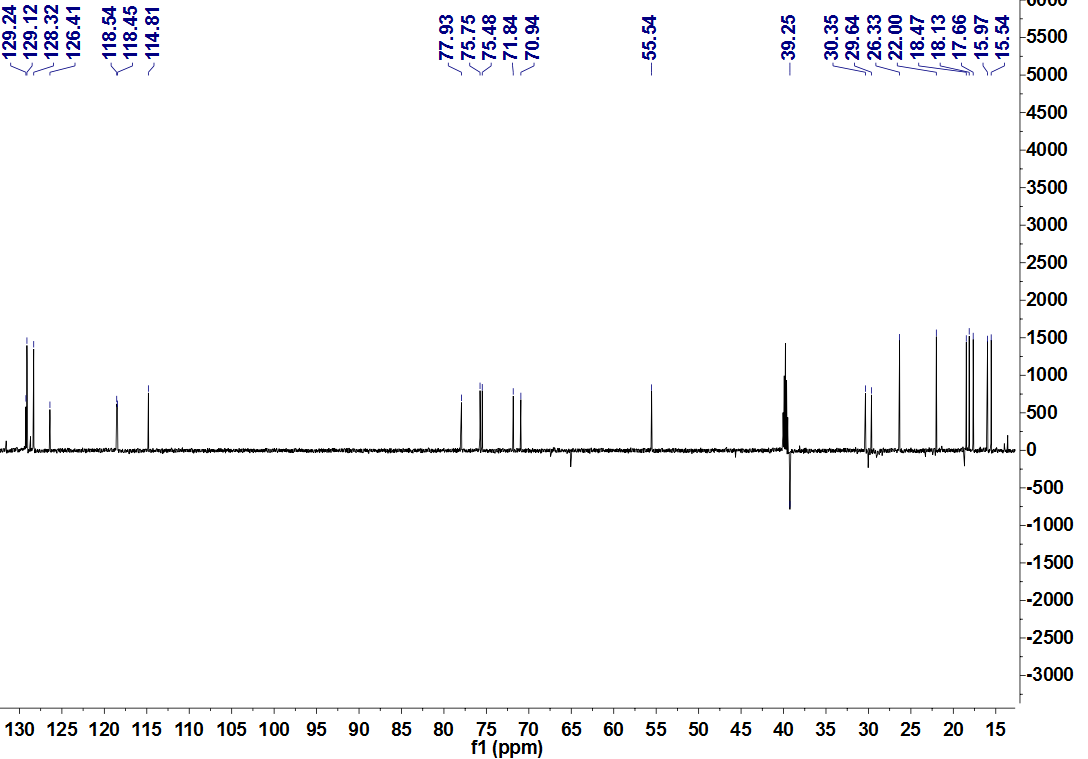
**

***d*.** ^1^H-^1^H COSY spectrum of compound **4**

**
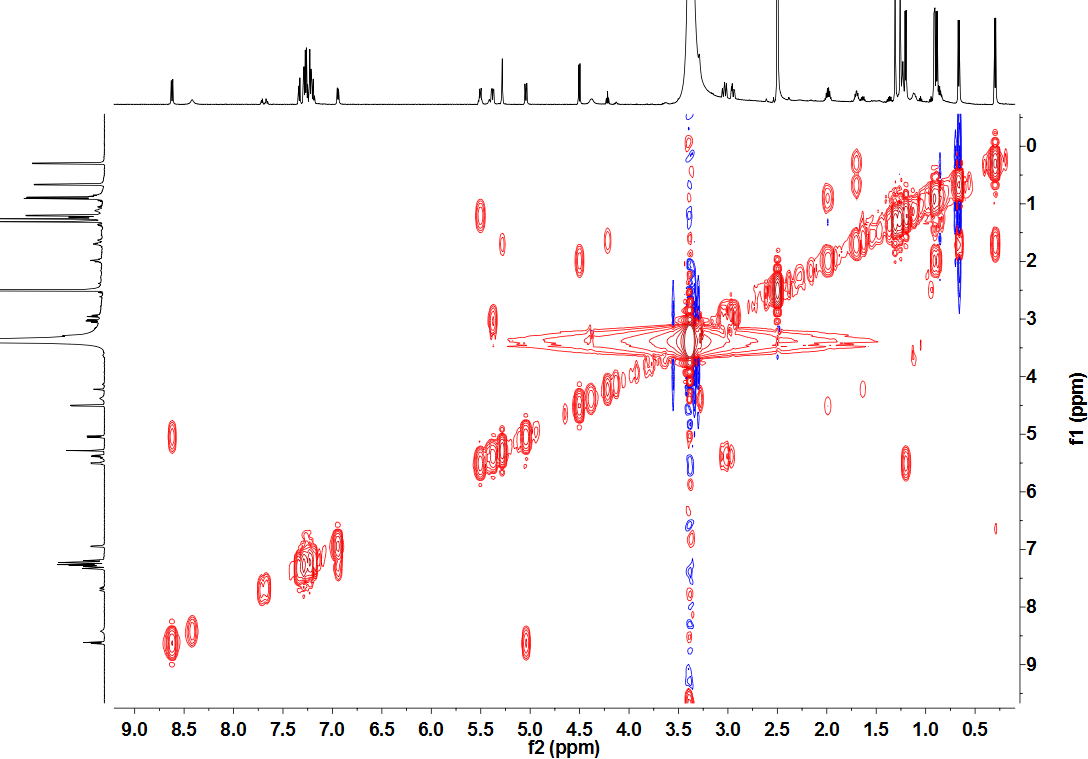
**

***e*.** HSQC spectrum of compound **4**

**
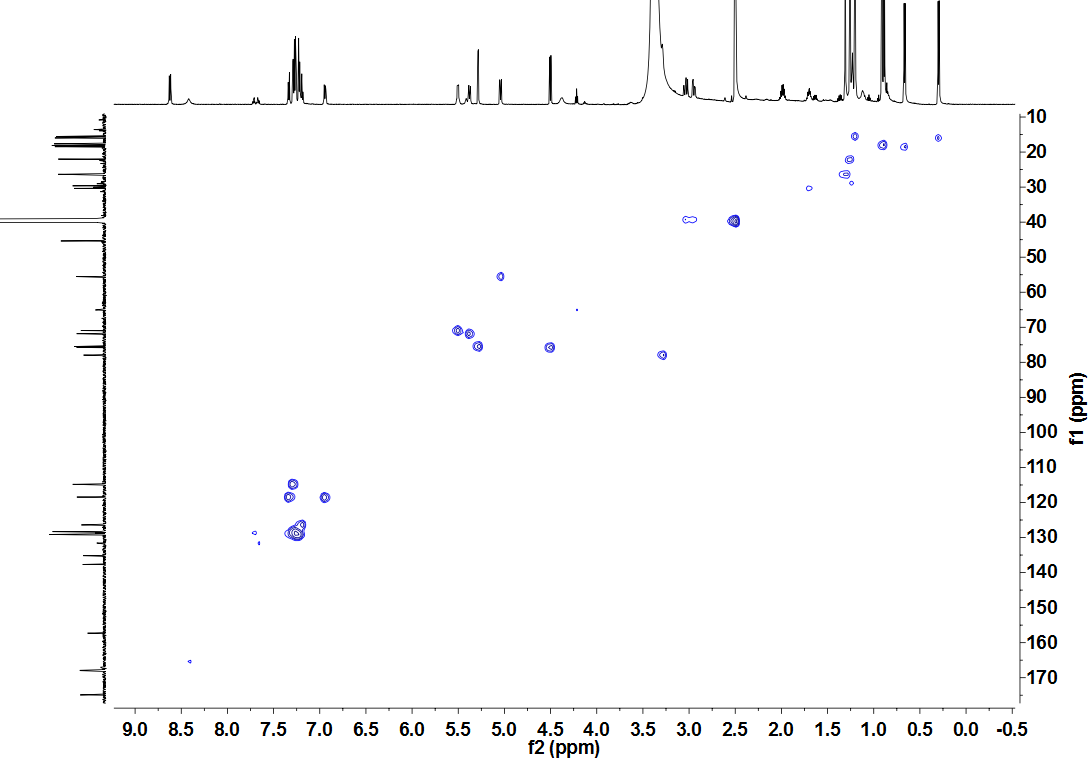
**

***f*.** HMBC spectrum of compound **4**

**
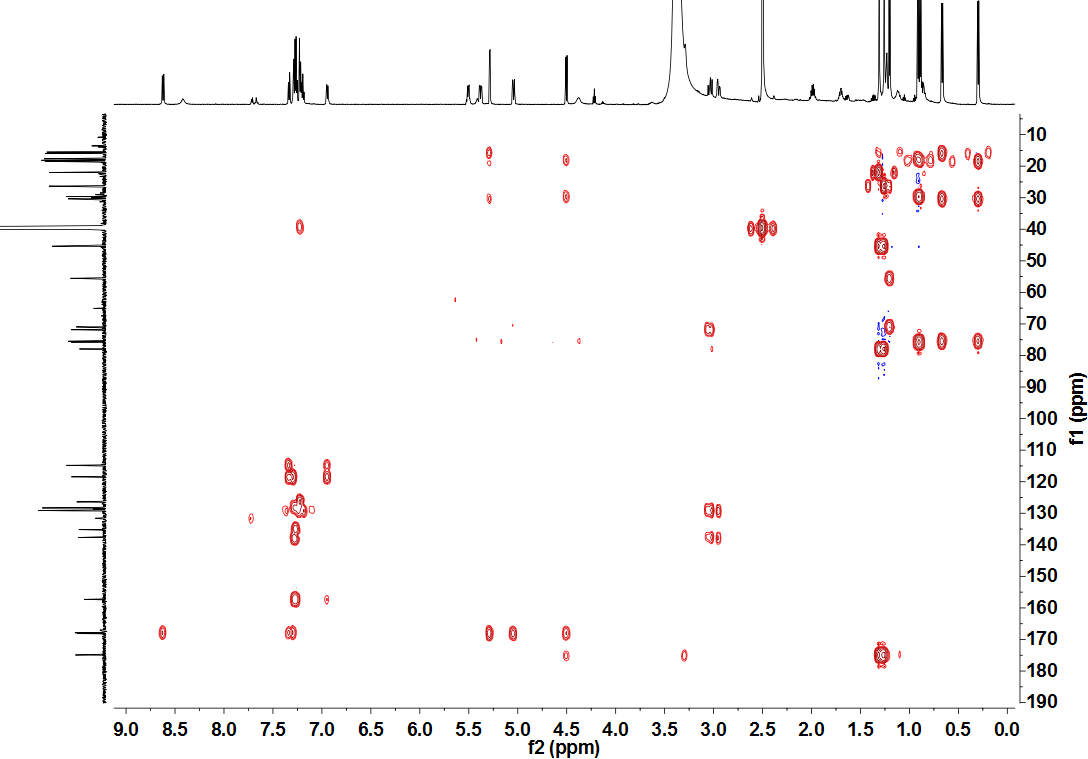
**

***g*.** NOESY spectrum of compound **4**

**
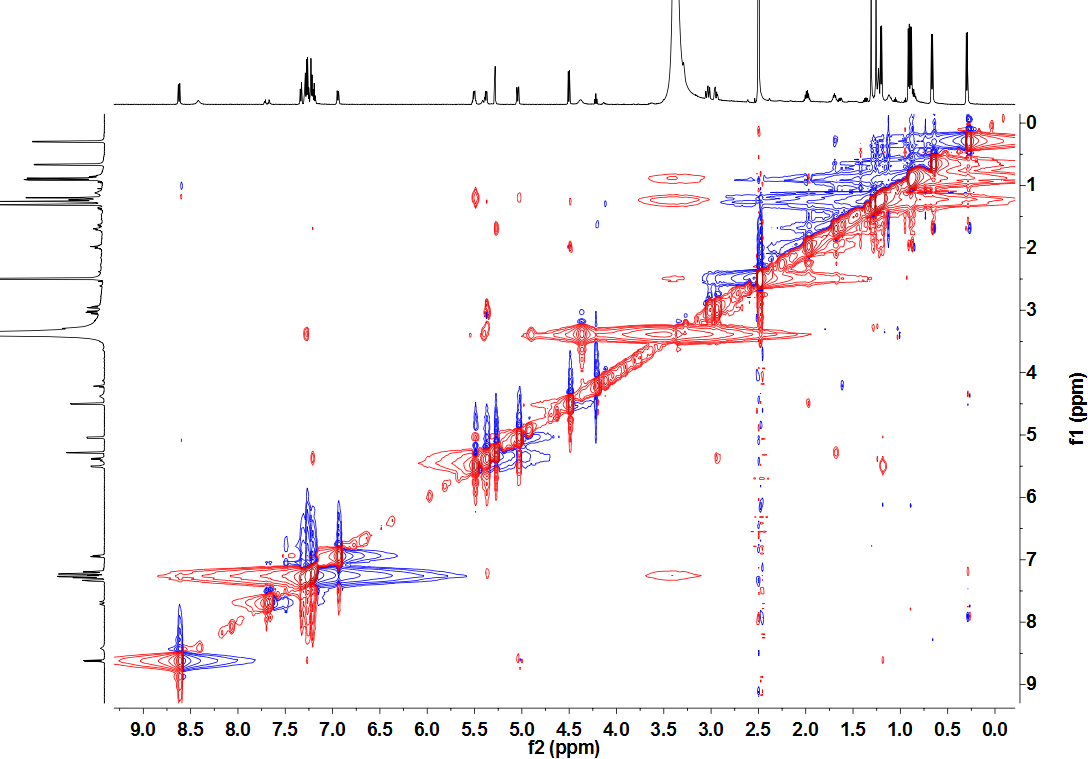
**
